# Shengjiang Xiexin decoction combined with vancomycin for *Clostridioides difficile* infection: impact of vancomycin dose-reduction strategy on gut microbiota homeostasis and recurrence risk

**DOI:** 10.3389/fcimb.2025.1740179

**Published:** 2026-02-11

**Authors:** Lin Zhu, Li-sheng Chen, Fu-zhi Ma, Jin-e Peng, Yu-bu Wang, Yu-qing Ma, Yue Xu, Yi Wang, Ayiman Yeerjiang, Cong-en Zhang, Zhi-jie Ma

**Affiliations:** 1 Department of Pharmacy, Beijing Ditan Hospital, Capital Medical University, Beijing, China; 2 Department of Pharmacy, Beijing Friendship Hospital, Capital Medical University, Beijing, China

**Keywords:** *Clostridioides difficile* infection (CDI), dosing strategy, gut microbiota, Shengjiang Xiexin decoction, vancomycin

## Abstract

Even though relapse rates for *Clostridioides difficile* infection (CDI) are high, vancomycin—a medication that targets *C. difficile* and works well during acute episodes—has gut microbiota-disrupting effects. The Chinese herbal formula Shengjiang Xiexin decoction (SXD) is helpful for microbial reversion, justifying the use of combination therapy. One often invoked tactic in such bundles of care is to minimize patients’ antibiotic exposure. In this work, we challenged this paradigm in a murine CDI model. Divergently with regard to the dose deescalation (low-dose vs. short-course), we found that it significantly undermined the synergy between the drugs. Despite the resolution of acute symptoms by all combination regimens containing SXD, deescalation strategies (CDR2 and CDR3) resulted in significantly worse relapse, enhanced inflammation and sustained gut dysbiosis. Conversely, only the CDR1 regimen with SXD co-treatment resulted in a full recovery of gut microbiota alpha-diversity and long-term ecological stability, associated with a better shift of metabolic pathways. Accordingly, our key finding is that standard vancomycin dosing is necessary for this therapeutic synergy to be realized and that dose deescalation blunts it and raises the risk of relapse. Such evidence invites a more sophisticated antibiotic stewardship approach with concomitant treatments, favoring preservation of synergistic effect over simplistic dose reduction.

## 1 Introduction

*Clostridioides difficile* is a Gram-positive, anaerobic, spore-forming bacterium ubiquitous in the environment and human gut; its secreted exotoxins cause severe intestinal diseases (Al-Zahrani, 2023). Normal gut microbiota is known to play an effective role in suppressing the overgrowth of *C. difficile* (Mileto et al., 2019). However, frequent use of antibiotics, especially broad-spectrum antibiotics, disrupts the balance of normal gastrointestinal microbiota and allows invasion by resistant *C. difficile*, which result in *C. difficile* Infection (CDI) (Haak et al., 2019; Spigaglia, 2022). CDI causes up to 25% of all healthcare-associated infectious diarrhea and can present with mild diarrhea to severe pseudomembranous colitis (PMC). Its great recurrence brings a serious public health concern worldwide (Poutanen and Simor, 2004; Xu et al., 2021; Guh et al., 2020; Boven et al., 2023). The underlying pathogenesis primarily results from direct cytotoxic effects of toxins A and B on colonic epithelial cells , causing inflammation and tissue damage (Pothoulakis, 2000; Voth and Ballard, 2005).

Present CDI clinical therapy is mainly based on antibiotics, which include vancomycin (Baunwall et al., 2022; Stevens et al., 2017). However, vancomycin does not kill *C. difficile* spores and may perturb the microbiota in the gut during treatment, which may damage gut barrier function and can lead to relapse of infection (reported to be 20% following a single course of treatment (Isaac et al., 2017; Aslam et al., 2005). Regarding vancomycin dosage and duration, clinical practice and guidelines emphasize balancing rapid bacterial clearance, recurrence risk, and gut microbiota reconstruction (Marchandin et al., 2023). For instance, recurrent CDI treatment increasingly favors “tapered/pulsed regimens”, typically starting with 125 mg QID then gradually reducing frequency over weeks, aiming for intermittent spore suppression while lessening continuous burden on commensal flora (Johnson et al., 2021; Bishop and Tiruvoipati, 2023; Shoff et al., 2022). For initial episodes, “dose escalation” is not encouraged: multiple studies show 125 mg QID achieves comparable clinical cure and recurrence rates to higher doses (Chiu et al., 2019; Ereshefsky et al., 2015). Furthermore, when systemic antibiotics are required for non-CDI indications, low-dose vancomycin (secondary prophylaxis, 125 mg QD) has shown some benefit in reducing recurrence, offering a viable path to minimize total exposure (Zhang et al., 2019; Keating et al., 2025). Hence, there remains a considerable unmet requirement for safe and effective treatments against CDI with low recurrence rates.

Traditional Chinese Medicine (TCM), with its rich experience in infectious disease management and holistic regulatory approach, offers a unique perspective for comprehensive CDI management. TCM theory categorizes CDI under “diarrhea” and “dysentery.” Its multi-target mechanisms are expected to suppress pathogens, alleviate inflammation, promote intestinal mucosal repair, and regulate gut microbiota balance (Cui et al., 2023; Gao et al., 2019). Shengjiang Xiexin decoction (SXD), from Shanghan Lun, is traditionally used for gastrointestinal ailments and to stop diarrhea and vomiting (Deng et al., 2020). Modern pharmacological research indicates SXD protects intestinal cells, promotes mucosal repair, and improves the microenvironment by regulating intestinal metabolites, thereby maintaining gut health (Cui et al., 2023; Deng et al., 2017).

Given vancomycin’s efficacy in functions and the potential of SXD to restore gut homeostasis and reduce pathogen recurrences, we speculate that the combination of these two approaches would offer synergistic effects for improved therapeutic benefits. These patterns of integrated Chinese and Western medicine have proved successful in other areas as well (Li et al., 2021; Zhou et al., 2022). Our preliminary evidence also supported this in the first place: combined application of SXD and vancomycin was able to rapidly relieve CDI symptoms when used during recovery, it was no less effective at preventing recurrence and even performed better than either drug alone, exerting complementary or synergistic effects (Yu et al., 2024). Based on these findings, we speculate that the underlying mechanism of this synergistic effect may stem from the complementary modes of action of the two therapies: vancomycin primarily controls acute infection by directly killing *Clostridioides difficile* vegetative cells, whereas SXD may improve the intestinal environment by modulating gut microecological balance and promoting mucosal barrier repair. This complementary strategy of “bacterial eradication” and “microecological regulation” theoretically targets two key aspects of CDI pathogenesis—pathogen overgrowth and intestinal microenvironment disruption—potentially leading to clinical outcomes superior to those achieved with monotherapy.

However, vancomycin’s disruption of gut microbiota remains a concern; long-term or high-dose use may increase resistance risk and impair microbial reconstruction. Given the potential of Shengjiang Xiexin decoction (SXD) to effectively restore gut microbiota and reduce recurrence, the combination of SXD and vancomycin offers a new therapeutic strategy for CDI. Nevertheless, the optimal vancomycin dosage within this combination therapy remains unclear. We hypothesize that the synergistic efficacy of SXD combined with vancomycin may be highly dependent on the vancomycin dosage. This study therefore aims to determine the optimal dosage required for this synergy and to specifically investigate whether inappropriate dose de-escalation (either low-dose or short-course) would disrupt such synergistic effects and consequently increase the risk of relapse. Our goal is to provide a more precise and personalized drug optimization strategy and scientific basis for the clinical treatment of CDI with SXD combined with vancomycin.

## 2 Materials and methods

### 2.1 Chemicals and reagents

Vancomycin (Batch no. S17059), Metronidazole (S17079), Colistin (S17057), Gentamicin (V30125), and Clindamycin (B61748) were purchased from Shanghai Yuanye Bio-Technology Co., Ltd. The RNAprep Pure Tissue Total RNA Isolation Kit (DP431) was obtained from TIANGEN. The Fecal DNA Extraction Kit (D4015010000D01V051) was supplied by OMEGA. Premix ExTaq™ (1927827100), probes, and primers (AK81837A) were acquired from Sangon Biotech (Shanghai) Co., Ltd. First-Strand Synthesis Master Mix and cDNA reverse transcription kits (0202122021) were from Lamborlyde Biotechnology. qPCR SYBR Green Master Mix (Low Rox Plus) (H8216980) was purchased from YESEN. Pentobarbital sodium (P3761) was obtained from Sigma-Aldrich Co., Ltd. The *Clostridium difficile* 027 (BI/NAP1/027) strain originates from the Department of Clinical Laboratory, Beijing Friendship Hospital, Capital Medical University.

### 2.2 Preparation of experimental drugs

Zingiberis Rizoma Recens (*Zingiber officinale* Rosc., batch number: 20110404) was sourced from Beijing Renwei Herbal Pieces Factory, China. Zingiberis Rhizoma (*Zingiber officinale* Rosc., batch number: 20093003) and Jujubae Fructus (*Ziziphus jujuba Mill*., batch number: 201105005) were from Beijing Qiancao Chinese Herbal Pieces Co., Ltd. Ginseng Radix et Rhizoma (*Panax ginseng* C. A. Mey., batch number: 1906005) was obtained from Beijing Tongchuntang Hospital of Traditional Chinese Medicine. Glycyrrhizae Radix et Rhizoma Praeparata Cum Melle (*Glycyrrhiza uralensis* Fisch., batch number: 200516) was provided by Beijing Yanbei Herbal Pieces Factory. Coptidis Rhizoma (*Coptis chinensis* Franch., batch number: 1912205) and Scutellariae Radix (*Scutellaria baicalensis* Georgi, batch number: 1912204) were supplied by Beijing Shuangqiao Yanjing Chinese Herbal Pieces Factory. Pinelliae Rhizoma Praeparatum (*Pinellia ternate* (Thunb.) Breit., batch number: 1231144) was from Beijing Tongrentang Co., Ltd., China.

SXD was prepared according to established methods (Cui et al., 2023). The formulation contains eight herbal components: Zingiberis Rhizoma Recens 12 g, Glycyrrhizae Radix et Rhizoma Praeparata cum Melle 9 g, Ginseng Radix et Rhizoma 9 g, Zingiberis Rhizoma 3 g, Scutellariae Radix 9 g, Pinelliae Rhizoma 9 g, Coptidis Rhizoma 3 g, and Jujubae Fructus 9 g. The herbs are first immersed in pure water at a solid-to-liquid ratio of 1:8 (w/v) for 30 minutes, followed by decoction for 30 minutes. The resulting filtrate is collected, and the residue is boiled again with water at a ratio of 1:6 (w/v) for an additional 30 minutes. The two filtrates are then combined and concentrated to a specified concentration. The prepared solution was stored at 4°C until use.

### 2.3 Experimental animals and study design

Eighty-four male C57BL/6 mice aged 5 weeks and weighing 20 ± 2 g were obtained from Beijing SPF Biotechnology Co., Ltd. (Experimental Animal Production License No.: SCXK (Jing) 2019-0010), an accredited animal supplier. The experiment procedure was reviewed and approved by the Institution of Animal Care and Use Committee (IACUC); in addition, written consent for experimental use had been provided by supplier for animal. The animals were maintained under controlled conditions (20–22°C, 12:12 h light: dark) in their home cage. Food and water were available ad libitum in the form of regular rodent chow and normal tap water. All animal procedures were in accordance with the standards of the Animal Ethics Committee of Beijing Friendship Hospital, Capital Medical University. The C57BL/6 mice were randomly assigned to seven groups, with 12 animals per group: Control (Con) group, Model (Mod) group, Vancomycin (Van) group, Shengjiang Xiexin Decoction (SXD) group, Combined Drug Regimen 1 (CDR1) group, Combined Drug Regimen 2 (CDR2) group, and Combined Drug Regimen 3 (CDR3) group.

**Figure 1:**
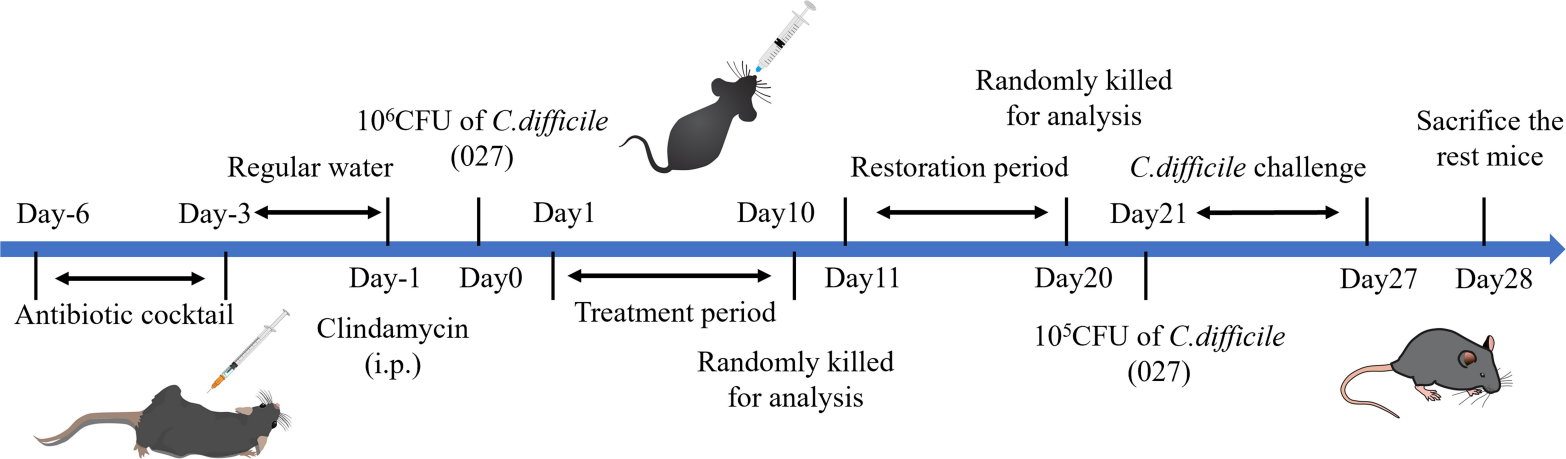
Experimental flowchart. This figure outlines the experimental design, structured into several distinct phases: antibiotic pre-treatment, *Clostridioides difficile* infection modeling, a 10-day drug intervention, a subsequent 10-day recovery period, and a 7-day re-challenge phase simulating clinical recurrence.

The entire experiment spanned 34 days, with the experimental procedure and dosing regimens detailed in **Table 1** and **Figure 1**. The Control and Model groups received an equivalent volume of physiological saline. The experimental animal treatment process was as follows: Except for the Con group, which maintained a normal diet and water, all other groups of mice were initially given a mixed antibiotic solution ad libitum (Vancomycin: 0.0225 mg/mL; Metronidazole: 0.1075 mg/mL; Colistin: 425 U/mL; Gentamicin: 0.0175 mg/mL) for 3 consecutive days. This was followed by a 3-day period of normal drinking water. On the second day of normal water consumption, all groups except the Con group received a single intraperitoneal (i.p.) injection of 10 mg/kg clindamycin for pretreatment. After the modeling period, an infection model was established in all groups (except Con) by oral gavage with 1×10^6^ CFU/mL *Clostridioides difficile* spore suspension. A 10-day interval of drug treatment followed. After drug administration, an observation period of 10 days was determined. After sample collection at the recovery stage, 4 mice in each group (except Con) were directed to a 7-day re-challenge with 1 ×10^5^ CFU/mL *C. difficile* spore suspension to mimic clinically relapse. During the course of the experiment, mice were observed daily for mental state, activity and fecal texture, whereas body weight was monitored at regular intervals. Sample collections. Specific time points were organized: During the treatment period (days 2, 5, 8 and 10) and recovery phase (days 11, 14, 17 and 20), fecal samples from mice were collected. At the end of the treatment period, four mice were euthanized for tissue collection. Most remaining mice were euthanized at the end of the recovery period, with four mice per group reserved for the re-challenge observation phase. During the re-challenge period, fecal samples continued to be collected (days 1, 3, 5, and 7). At the experiment’s conclusion, all remaining mice were euthanized, and final samples were collected. For euthanasia and tissue collection, mice were anesthetized via i.p. injection of pentobarbital sodium (50 mg/kg). Experimental procedures, including the collection of colon tissue and cecal contents for subsequent analysis, commenced after the loss of the righting reflex. Euthanasia was confirmed by an additional i.p. injection of pentobarbital sodium (200 mg/kg) after the procedures (Sugita et al., 2012).

**Table 1:** Dosing schedule and interventions.

Group	Treatment period		Restoration period
	Day 0-5	Day 6-10	Day 11-20
Con	Normal saline	Normal saline	No treatment
Mod	Normal saline	Normal saline	No treatment
Van	A(50 mg/kg Van)	A(50 mg/kg Van)	No treatment
SXD	B(16.4 g/kg SXD)	B(16.4 g/kg SXD)	No treatment
combination drug regimen (CDR) 1	A+B	A+B	No treatment
combination drug regimen (CDR) 2	A+B	B	No treatment
combination drug regimen (CDR) 3	0.5A+B	0.5A+B	No treatment

### 2.4 Assessment of clinical symptoms and fecal consistency

**Figure 2:**
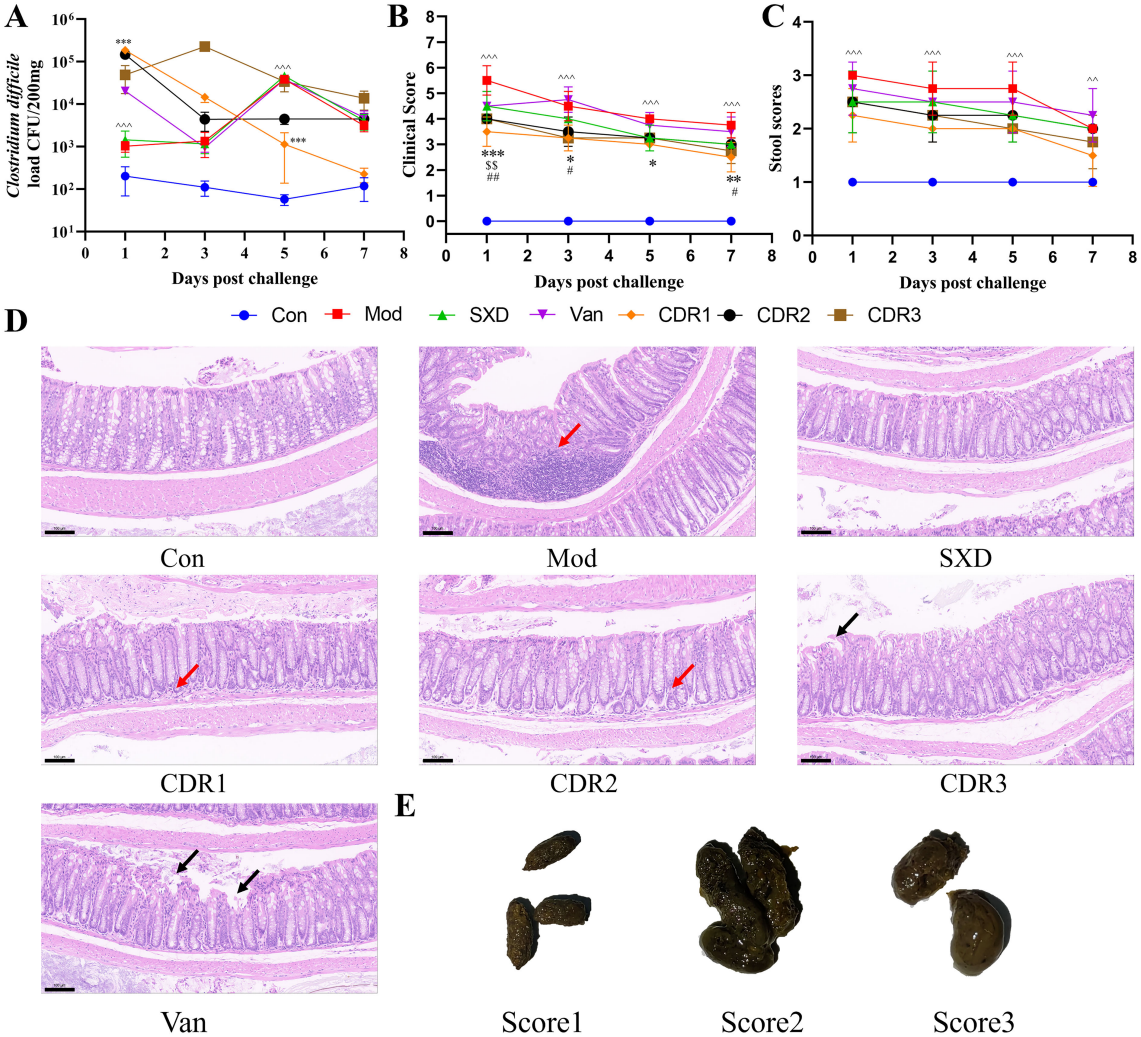
Improvement of pharmacodynamic parameters during the recurrence challenge phase. **(A)** *C. difficile* load; **(B)** clinical scores; **(C)** stool scores; **(D)** histopathological assessment of mouse colonic tissue (scale bar: 100 μm, 20×); **(E)** criteria for fecal scoring. Black arrows: shed mucosal epithelial cells; Red arrows: inflammatory cell infiltration. Data are presented as mean ± SD (n=4/group). Statistical comparisons were performed using one-way ANOVA. Statistical significance: compared with Con group, ^^P < 0.01, ^^^P < 0.001; compared with CDR1 group, *P < 0.05, **P < 0.01, ***P < 0.001; compared with CDR2 group, $$P < 0.01; compared with CDR3 group, #P < 0.05, ##P < 0.01.

During the whole period of experimentation, mice were closely monitored at 9:00 AM every day for general health conditions by measuring their mental state, activity and feces. Clinical symptoms of CDAD were assessed based on the criteria outlined in **Table 2**. Two factors, the state of mice and consistency in feces, were consistently observed and then scored according to the guidelines (Fachi et al., 2020). More specifically, fecal consistency was evaluated and categorized using a three-point visual grading scale (as depicted in **Figure 2E**): a score of 1 was assigned to formed, brown feces that retained their shape; semi-formed or soft, yellow feces that did not flow easily received a score of 2; and liquid, yellow feces that flowed easily were assigned a score of 3.

**Table 2:** Clinical score.

Category	Score			
	0	1	2	3
Activity	Normal	Alert/slow moving	Lethargic/shaky	Inactive unless prodded
Posture	Normal	Back slanted	Hunched	Hunched/nose down
Coat	Normal	Piloerection	Rough skin	Extremely ruffled/puff/ ungroomed
Diarrhea	Normal	Soft: stool/discolored(yellowish)	Wet stained tail/mucous±blood	Liquid/no stool (ileus)
Eyes/nose	Normal	Squinted/Partially closed	Squinted/discharge	Closed/discharge

### 2.5 RNA extraction and real-time quantitative PCR analysis

Total RNA was extracted from intestinal tissues using the RNAprep Pure Tissue Total RNA Isolation Kit (TIANGEN, DP431). Subsequently, RNA was reverse-transcribed into cDNA using a reverse transcription kit (Beijing LABLEAD Biotechnology Co., Ltd., 0202122021). The reverse transcription protocol involved an initial incubation at 37°C for 2 minutes, followed by 55°C for 15 minutes, 85°C for 5 minutes, and then storage at 4°C. Quantitative PCR reactions were performed in a 20 μL system containing 2 μL of cDNA template, 10 μL of SYBR Green Master Mix, 1 μL of forward primer, 1 μL of reverse primer, and 6 μL of Nuclease-Free Water. The amplification program was carried out in two steps: initial denaturation at 95°C for 5 min, then the cycles went as follows: 40 cycles of denaturation at 95°C for 10 seconds and annealing/extension at 60°C for 30 seconds. The expression of IL-1β, CXCL-1, Occludin, Claudin-1, Muc-2, TNF-α and ZO-1 in colon tissues was measured by RT-qPCR; the internal reference gene was GAPDH. All primer and probe sequences utilized are detailed in **Table 3**.

**Table 3:** Primer sequences for real-time quantitative PCR (qPCR).

Genes	Primer sequences
IL-1β	F: TGTGAAATGCCACCTTTTGA, R: GTCAAAGGTTTGGAAGCAG
CXCL-1	F: CTTGAAGGTGTTGCCCTCAG R: TGGGGACACCTTTTAGCATC
Occludin	F: TACTGTGTGGTTGATCCCCAG R: GATAATCATGAACCCCAGGAC
Muc-2	F: TCCCTGGCCTCTGTGATTACA R: AGTAGACCTTGGTGTAGGCA
ZO-1	F: CCACTCTTCCAGAACCGAAACCTG R: TTTCATGCTGGGCCTAAGTATCCC
TNF-α	F: CTCCAGGCGGTGCCTATGT R: GAAGAGCGTGGTGGCCC
Claudin-1	F: CCGGGCAGATACAGTGCAA R: TCTTCCAGGCACCTCATGC
GAPDH	F: GGCAAGGTCATCCCAGAGCTG R: ATCCACGACGGACACATTGGG

### 2.6 Fecal *Clostridioides difficile* load detection

Mice were individually moved to clean cages and fresh fecal pellets collected with sterile forceps at the appropriate time points, directly into 2 mL cryovials (approximately 200 mg or 6–8 pellets). These samples were immediately frozen in a bath of liquid nitrogen. Total DNA from mouse fecal samples was subsequently extracted strictly according to the manufacturer’s instructions for the Stool DNA Kit. A standard curve was generated through serial dilutions, and *Clostridioides difficile* load was quantified by relating Ct values to Log10 values of bacterial load. Real-time quantitative PCR (qPCR) was performed using specific primers and a TaqMan probe. The reaction conditions involved an initial denaturation at 95°C for 30 seconds, followed by 40 cycles of denaturation at 95°C for 3 seconds and annealing/extension at 50°C for 30 seconds. Absolute quantification was employed for the RT-qPCR analysis of mouse fecal samples.

### 2.7 Histological analysis

Fresh distal colon tissues were meticulously prepared using the “Swiss roll” technique and then fixed in 4% paraformaldehyde in a fume hood before paraffin embedding. After sectioning, the tissues were stained with Hematoxylin and Eosin (H&E). Pathological changes in the colon were observed under a microscope, and representative images were captured for subsequent analysis. Imaging assistance was provided by Wuhan Seville Biotechnology Co., Ltd.

### 2.8 Colon content 16S rRNA gene sequencing

Full-length 16S rRNA gene sequencing was performed to obtain comprehensive information on microbial taxonomy and diversity by targeting the V1-V9 regions through multiplex PCR amplification. Cecal content samples were collected, and DNA was extracted using a fecal DNA extraction kit. Library construction involved PCR amplification of the V3-V4 hypervariable regions. Following library preparation, single-molecule sequencing of the full-length 16S amplicons was conducted on the PacBio sequencing platform. After detection, multiplex PCR amplification and purification were performed, and samples were loaded for sequencing. Post-sequencing, raw paired-end data were assembled using overlap reads and subjected to quality control to yield high-quality Clean Data. To minimize noise, raw sequencing data underwent rigorous filtering, and the DADA2 (Divisive Amplicon Denoising Algorithm) was utilized for further denoising to extract more accurate sequence information and improve data precision, resulting in Effective Tags. Based on these effective tags, OTU (Operational Taxonomic Unit) clustering/denoising and species classification analyses were performed to construct species abundance profiles and to analyze OTU abundance, diversity indices, and community structure. Furthermore, clustering analysis, statistical comparisons, and correlation analysis of OTU and species composition were conducted to identify inter-sample species differences. Advanced bioinformatics analyses, including alpha (α) diversity analysis, beta (β) diversity analysis, species taxonomic identification, and functional prediction, were also performed on the generated sequence data.

### 2.9 Statistical methods

All quantitative data are presented as the mean ± standard deviation (SD). Statistical analyses were performed using SPSS 26.0 software, and graphs were generated with GraphPad Prism 9.0. For comparisons between two normally distributed samples, Student’s t-test was used. One-way ANOVA was employed to determine statistical significance for more than two independent samples following a normal distribution. When data did not conform to a normal distribution or exhibited unequal variances, the non-parametric Kruskal-Wallis rank-sum test was applied. A P-value of less than 0.05 (P < 0.05) was considered statistically significant.

## 3 Results

### 3.1 Conventional and short-course combined regimens yield optimal therapeutic outcomes

**Figure 3:**
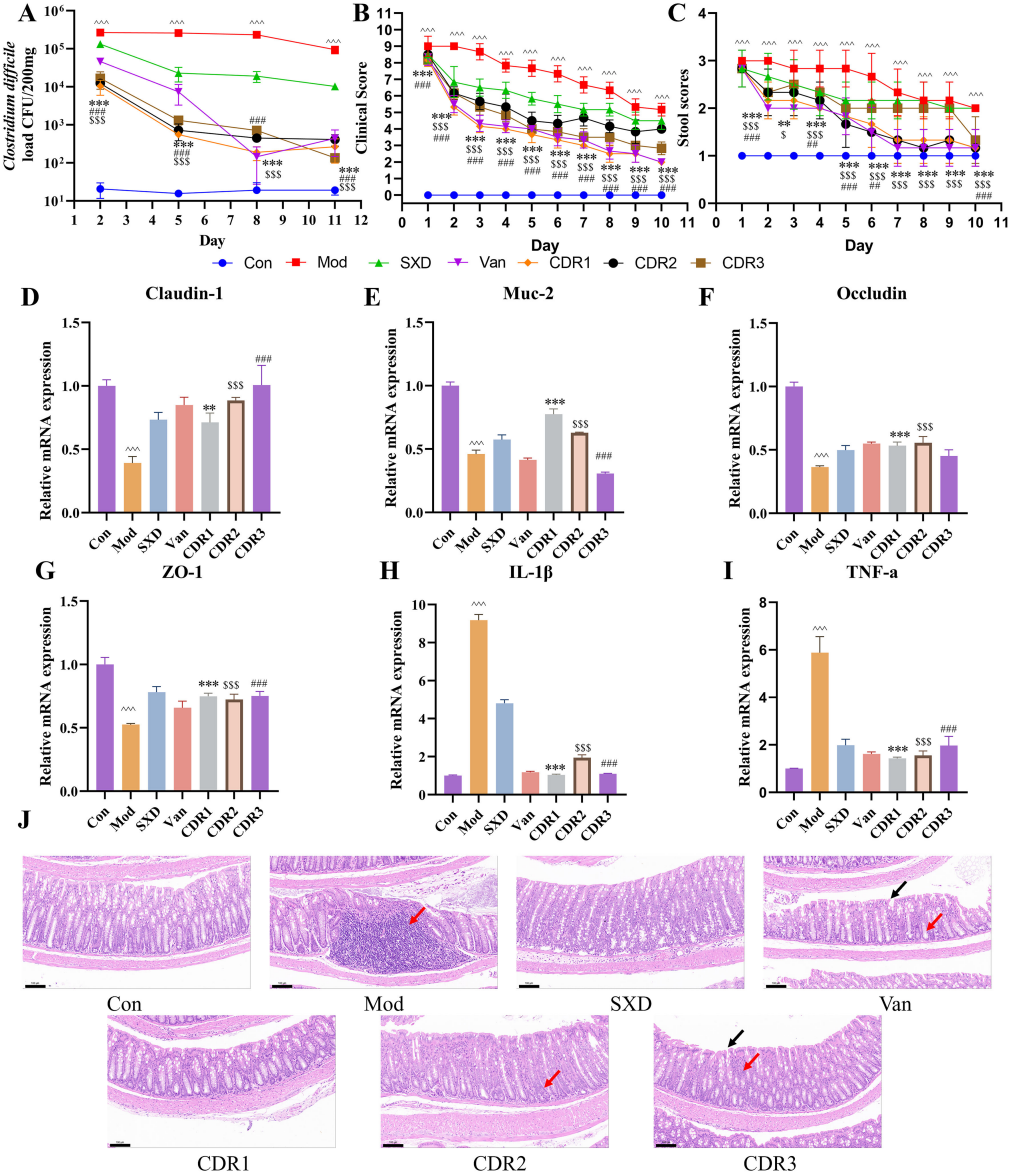
Improvement of pharmacodynamic parameters during the treatment phase. **(A)** *C. difficile* load, **(B)** clinical scores, and **(C)** stool scores. RT-qPCR analysis of mRNA expression of Claudin-1 **(D)**, Muc-2 **(E)**, Occludin **(F)**, ZO-1 **(G)**, IL-1β **(H)**, and TNF-α **(I)** in colonic tissues post-treatment. **(J)** histopathological analysis of mouse colonic tissues (Scale bar: 100 μm; magnification: 20×). Black arrows: shed mucosal epithelial cells; Red arrows: inflammatory cell infiltration. Data are presented as mean ± SD(n=4/group). Statistical comparisons were performed using one-way ANOVA. Statistical significance: compared with Con group, ^^^P < 0.001; compared with CDR1 group, **P < 0.01, ***P < 0.001; compared with CDR2 group, $P < 0.05, $$$P < 0.001; compared with CDR3 group, ##P < 0.01, ###P < 0.001.

For evaluating the efficiency of treatment, we analyzed firstly clinical symptoms and bacterial burden. All of the combination treatments (CDR1, CDR2, and CDR3) were more effective in mitigating CDI than were those with vancomycin alone (Van monotherapy), quickly improving clinical scores and fecal consistency to a comparable level with Van treatment (**Figures 3B, C**). Conversely, both treatment groups experienced durable and prompt reductions in fecal *C. difficile* load, consistent with the killing kinetics of the standard Van regimen (**Figure 3A**). By comparison, monotherapy with SXD performed relatively weak on clinical outcomes and had markedly less bacterial eradication than Van and the combination groups. These results establish that the combination therapies are as effective as vancomycin in controlling the acute infection.

The primary advantage of the combination therapies was their profound effect on restoring gut health. The three regimens all remarkably increased the mRNA levels of tight junction proteins (ZO-1, Claudin-1, Occludin) and attenuated critical inflammatory cytokines (TNF-α, IL-1β) (**Figures 3D–I**). Our molecular results were supported by our histopathological analysis, which demonstrated that combination-treated groups displayed more profound attenuation of inflammation and obvious epithelial repairing (**Figure 3J**). It is interesting to note that extracts from the CDR1 were shown to be significantly effective and also showed significant recovery of Muc-2 protein. In contrast, monotherapies failed to induce tissue repair. The bactericidal effect against the Van group could not avoid a remarkable inflammatory infiltration and epithelial injury. The SXD group only generated a certain degree of protection of the mucosa, but to overcome the etiological results.

In summary, the combination regimens, particularly CDR1, deliver a superior therapeutic outcome by addressing both the pathogen and the host pathology. They uniquely pair the potent bactericidal activity of vancomycin with the robust gut barrier restoration and anti-inflammatory effects of SXD. This dual action makes them more effective than either monotherapy alone. Furthermore, the other two combination regimens (CDR2 and CDR3) demonstrated a certain level of therapeutic effect during the acute treatment phase. Although their pathogen clearance efficacy was slightly inferior to that of CDR1, achieving this outcome with a halved dose of vancomycin highlights their potential clinical value. It is important to note that a comprehensive evaluation of these different treatment regimens requires an integrated analysis incorporating data from subsequent phases and the dynamic changes in the gut microbiota. While the CDR1 regimen exhibited the most comprehensive restorative effects on the gut, the robust performance of CDR2 in the acute phase is equally noteworthy. Specifically, its ability to maintain considerable therapeutic efficacy with a shorter course of vancomycin defines its advantage as a “potential alternative for acute-phase treatment in selected patient populations”—rather than a universally “safer option” for all patients—offering a targeted clinical choice for specific scenarios.

### 3.2 Conventional and low-dose therapies most effectively restore microbial diversity and composition

**Figure 4:**
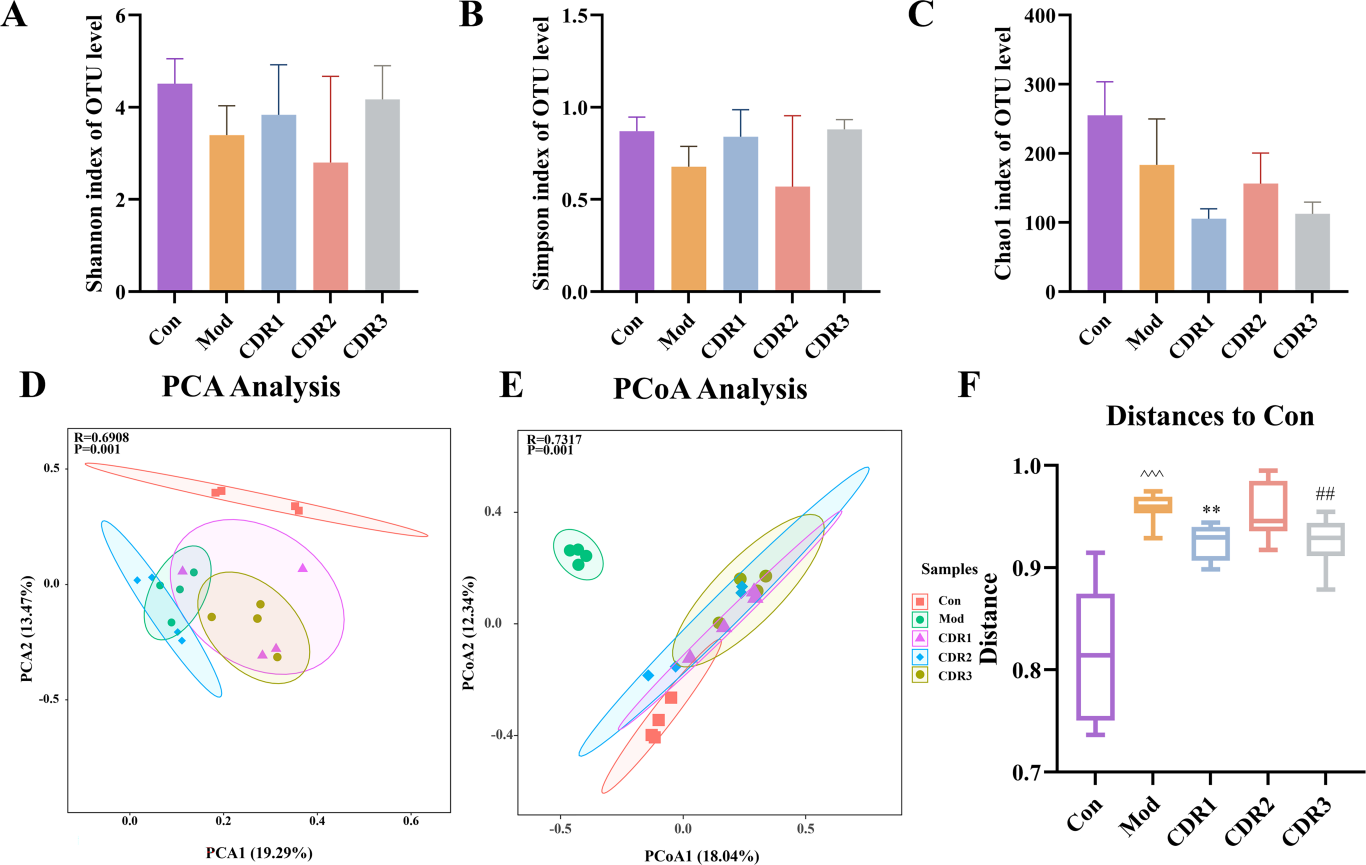
Analysis of gut microbiota diversity in mice during the treatment period. **(A-C)** Alpha diversity metrics: **(A)** Shannon Index, **(B)** Simpson Index, and **(C)** Chao1 Index. **(D-E)** Beta diversity analysis: **(D)** Principal Component Analysis (PCA) plot and **(E)** Principal Coordinate Analysis (PCoA) plot based on Bray-Curtis dissimilarity. **(F)** Boxplot showing the Bray-Curtis distance between the Control group and other treatment groups, indicating community structure differences. Data are presented as mean ± SD(n=4/group). Statistical significance was determined by one-way ANOVA with Tukey's post hoc test (for **A-C, F**) or PERMANOVA (for **D, E**). Statistical significance: compared with Con group, ^^^P < 0.001; compared with CDR1 group, **P < 0.01; compared with CDR3 group, ##P < 0.01.

To investigate therapeutic mechanisms, we performed 16S rRNA sequencing. CDI induction significantly reduced alpha diversity (Shannon, Simpson, Chao1), which all combination therapies partially restored. Notably, among all treatment groups, CDR1 demonstrated the most substantial restoration, with its Chao1, Shannon, and Simpson indices showing significantly greater improvement than CDR2, CDR3, and monotherapy groups (**Figures 4A–C**). Beta diversity analysis (PCoA, PCA) confirmed that the microbial structure of the CDR1 group shifted most closely to that of the Control (Con) group, significantly outperforming other regimens in restoring the overall gut community structure (**Figures 4D–F**).

**Figure 5:**
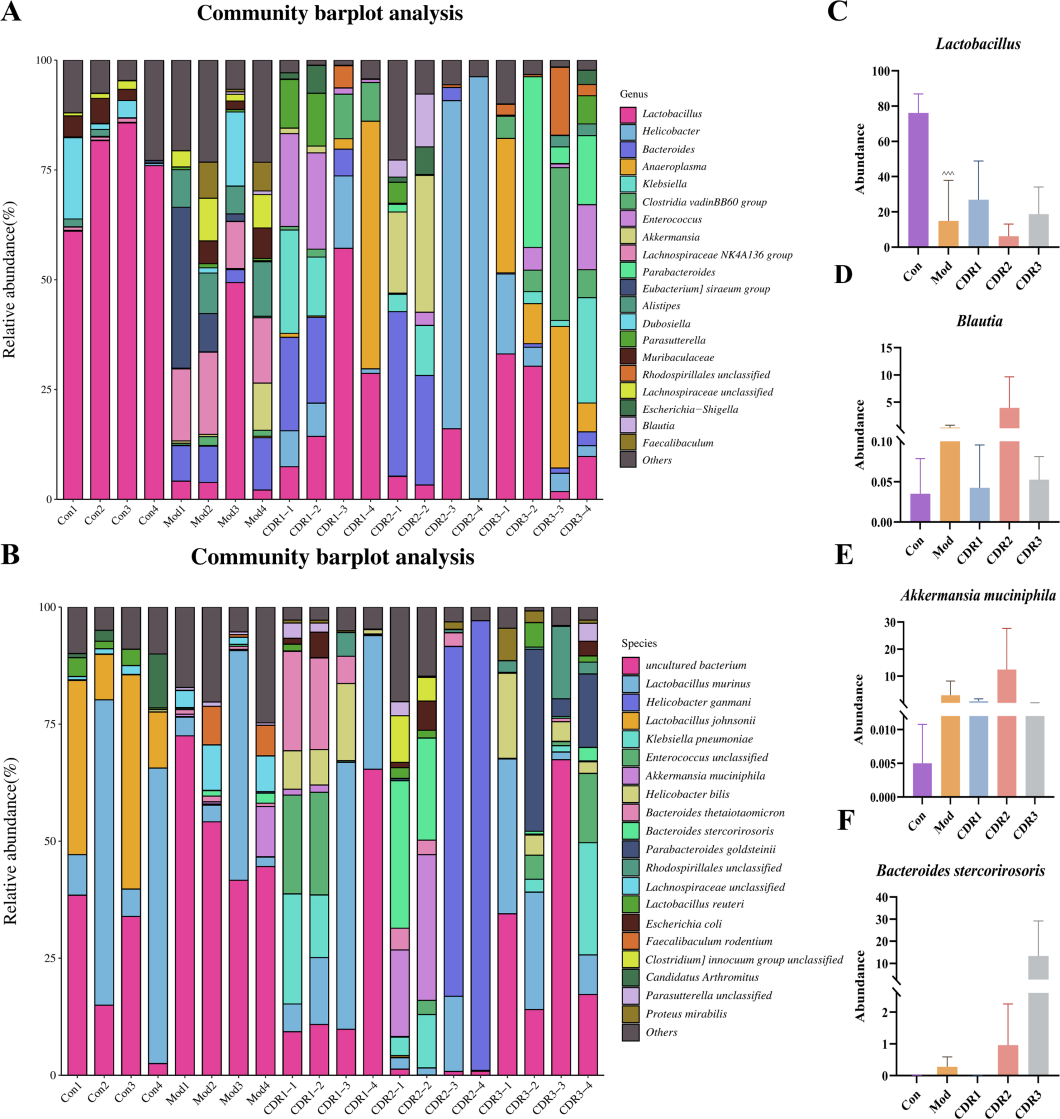
Gut microbial community composition at the genus and species levels during the treatment period. **(A)** Relative abundance of the top 20 most abundant genera. **(B)** Relative abundance of the top 20 most abundant species. **(C-F)** Relative abundance of key taxa: **(C)** Lactobacillus, **(D)** Blautia, **(E)** Akkermansia muciniphila, and **(F)** Bacteroides stercorirosoris. Data are presented as mean ± SD (n=4/group). Statistical significance was determined by one-way ANOVA followed by Tukey's post hoc test. Statistical significance: compared with Con group, ^^^P < 0.001.

At the taxonomic level, *Lactobacillus* was the dominant genus in control and most treated groups. Systematic comparison revealed that CDR1 achieved the most effective restoration of key genera, including *Blautia* and *Lactobacillus*, with relative abundances most closely resembling the Con group. At the species level, CDR1 exhibited comprehensive advantages in restoring beneficial species: it not only recovered *Akkermansia muciniphila* and *Bacteroides stercorirosoris* to near-normal levels, but also showed significantly superior restoration of *Bacteroides thetaiotaomicron*—a species crucial for intestinal barrier repair—compared to all other treatment groups (**Figures 5A–F**).

Analysis of the restorative effects during the treatment phase demonstrated that CDR1 most effectively shifted the microbial structure and key species—notably *Bacteroides thetaiotaomicron* (a species known for its role in intestinal barrier repair)—toward normal (Con) levels (**Supplementary Figures S1, 2**) (Wang et al., 2023). This superior microbial restoration by CDR1 was further corroborated by significantly enhanced colonization resistance against recurrent challenge, outperforming other treatment regimens.

In summary, the conventional combined regimen (CDR1) demonstrated comprehensive advantages over other therapeutic approaches in ameliorating gut dysbiosis, most effectively restoring microbial diversity, re-establishing key beneficial taxa, enhancing protective functional pathways, and ultimately providing superior colonization resistance.

### 3.3 Conventional combination therapy more effectively controls CDI recurrence

**Figure 6:**
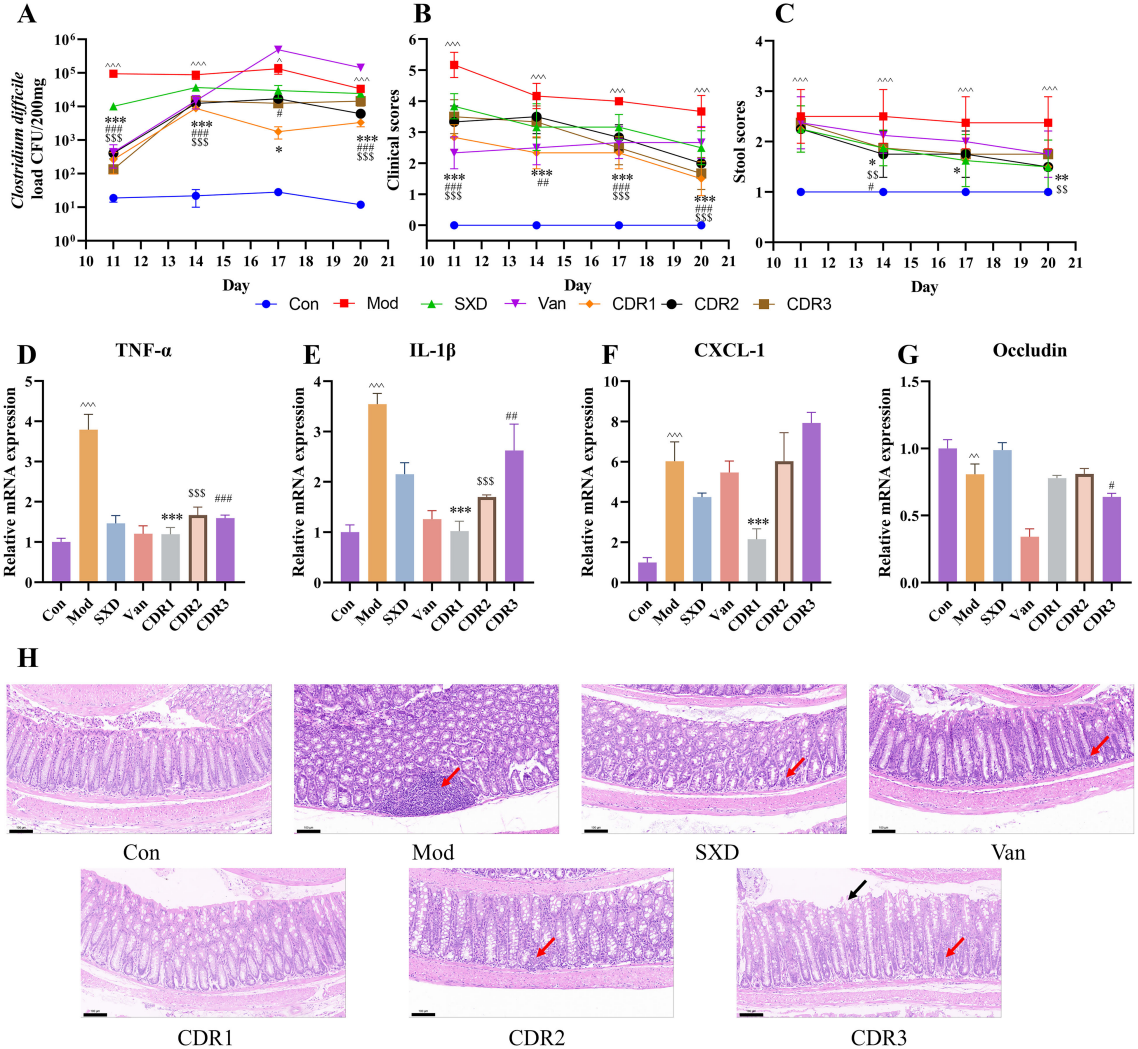
Improvement of pharmacodynamic parameters during the recovery phase. **(A)** *C. difficile* load, **(B)** clinical scores, and **(C)** stool scores. RT-qPCR analysis of mRNA expression of TNF-α **(D)**, IL-1β **(E)**, CXCL-1 **(F)**, and Occludin **(G)** in colonic tissues post-treatment. **(H)** histopathological analysis of mouse colonic tissues (Scale bar: 100 μm; magnification: 20×). Black arrows: shed mucosal epithelial cells; Red arrows: inflammatory cell infiltration. Data are presented as mean ± SD (n=4/group). Statistical comparisons were performed using one-way ANOVA. Statistical significance: compared with Con group, ^P < 0.05, ^^P < 0.01, ^^^P < 0.001; compared with CDR1 group, *P < 0.05, **P < 0.01, ***P < 0.001; compared with CDR2 group, $$P < 0.01, $$$P < 0.001; compared with CDR3 group, #P < 0.05, ##P < 0.01, ###P < 0.001.

In the recovery period, we considered treatment results on a long-term basis. The vancomycin (Van) monotherapy group had a severe bacterial rebound, and day 17 was higher than that of the model group. In contrast, among the combination regimens, all three (CDR1, CDR2 and CDR3) suppressed this rebound so that significantly lower levels of bacteria could be retained at a more constant level, indicating less pronounced recurrence (**Figure 6A**). CDR1 The CDR1 group was the best in all comparisons, maintaining the lowest *C. difficile* burden after treatment. These microbiologic results were in agreement with clinical observations. Mice in the combination regimens had both more pronounced and sustained improvement of their clinical scores and stool consistency compared to the Mod group (P<0.05 - P<0.001), with CDR1 and particularly CDR3 most effectively restoring normal stool form (**Figures 6B, C**).

We then evaluated the long-term effects on gut health. Histopathological analysis showed ongoing healing with better-organized intestinal cells and an intact barrier in the CDR1 group compared to all other groups when evaluating the percentage of shallow lesions (**Figure 6H**). By contrast, the CDR2 and CDR3 groups, as well as both Van monotherapy and SXD monotherapy groups, still displayed apparent inflammatory infiltration and epithelial damage.

Molecular analysis shed more light on these findings. Both the CDR1 and CDR2 regimens sustained low post-treatment levels of the inflammatory cytokines TNF-α and IL-1β (P<0.001 vs. Mod group). While the CDR2 group showed a strong recovery in the expression of tight junction proteins, the superior histological outcome of the CDR1 group suggests it conferred the most comprehensive and durable protection (**Figures 6D–G**). The monotherapies showed split effects: the Van group was more effective at reducing inflammatory markers, whereas the SXD group was superior in maintaining intestinal barrier components.

In summary, during the critical recovery period, the combination therapies—and particularly CDR1—provided a more holistic and effective outcome than monotherapies by simultaneously controlling bacterial recurrence, suppressing inflammation, and promoting durable structural healing of the intestinal mucosa.

### 3.4 Conventional combination and low-dose combination effectively improve gut microbiota dysbiosis in CDI mice during the recovery phase

**Figure 7:**
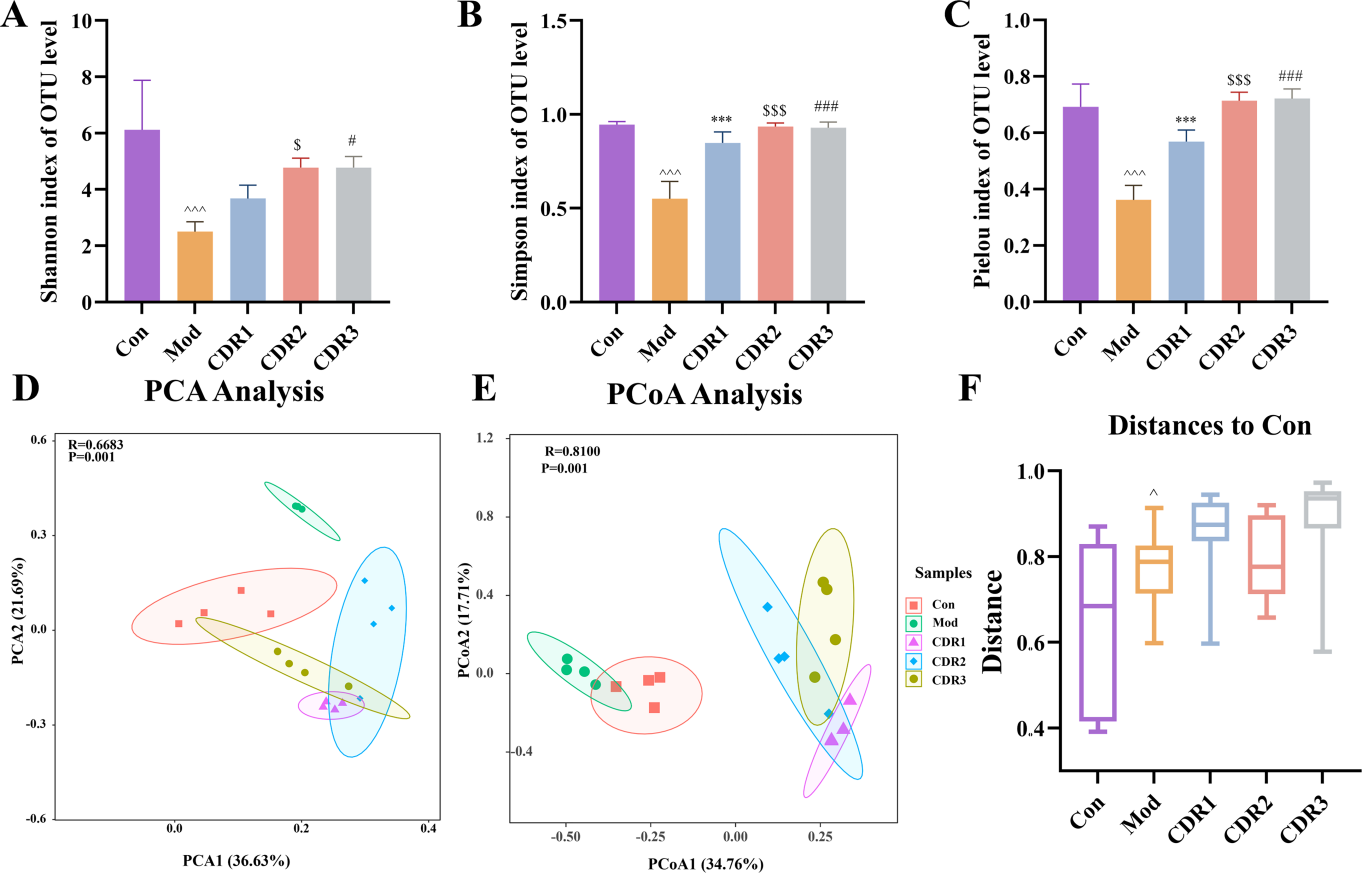
Diversity analysis of gut microbiota in mice during the recovery period. A-C Alpha Diversity Analysis. **(A)** Shannon Index, **(B)** Simpson Index, **(C)** Pielou Index, D-E Beta Diversity Analysis **(D)** PCA Analysis, **(E)** PCoA Analysis, **(F)** Boxplot showing the Bray-Curtis distance between the Control group and other treatment groups, indicating community structure differences. Data are presented as mean ± SD(n=4/group). Statistical significance was determined by one-way ANOVA with Tukey's post hoc test (for **A-C, F**) or PERMANOVA (for **D, E**). Statistical significance: compared with Con group, ^P < 0.05, ^^^P < 0.001; compared with CDR1 group, ***P < 0.001; compared with CDR2 group, $P < 0.05, $$$P < 0.001; compared with CDR3 group, #P < 0.05, ###P < 0.001.

The diversity of mouse gut microbiota in the recovery period was assessed. To evaluate alpha diversity, Important indexes were investigated, contained Simpson, Shannon and Pielou. Compared with the Con group, the Simpson and Shannon indices results after recovery reflected that there was a remarkable difference between the Mod and Con groups (P < 0.05, P < 0.01, or P < 0.001), as well as the evaluation of the Pielou index after restoration. This indicates that the gut microbiome in untreated CDI mice did not recover spontaneously. All combination groups, however, tended towards normalization with indices close to those of the Con group. Notably, among all treatment regimens, CDR1 demonstrated the most significant restoration in alpha diversity indices, outperforming both CDR2 and CDR3 as well as monotherapy groups (**Figures 7A–C**).

To assess differences in microbial community composition, beta diversity analysis was performed. Principal coordinate analysis (PCoA) and principal component analysis (PCA) revealed significant differences between the Con and Mod groups, indicating substantial alterations in the gut microbiota following CDI (**Figures 7D, E**). Boxplots based on Bray-Curtis dissimilarity further illustrated the community structure differences between the treatment and control groups. After treatment and recovery, while both CDR1 and CDR3 groups showed proximity to the Con group, CDR1 exhibited the closest alignment with the normal microbial structure, demonstrating superior efficacy over all other treatment groups (**Figure 7F**).

**Figure 8:**
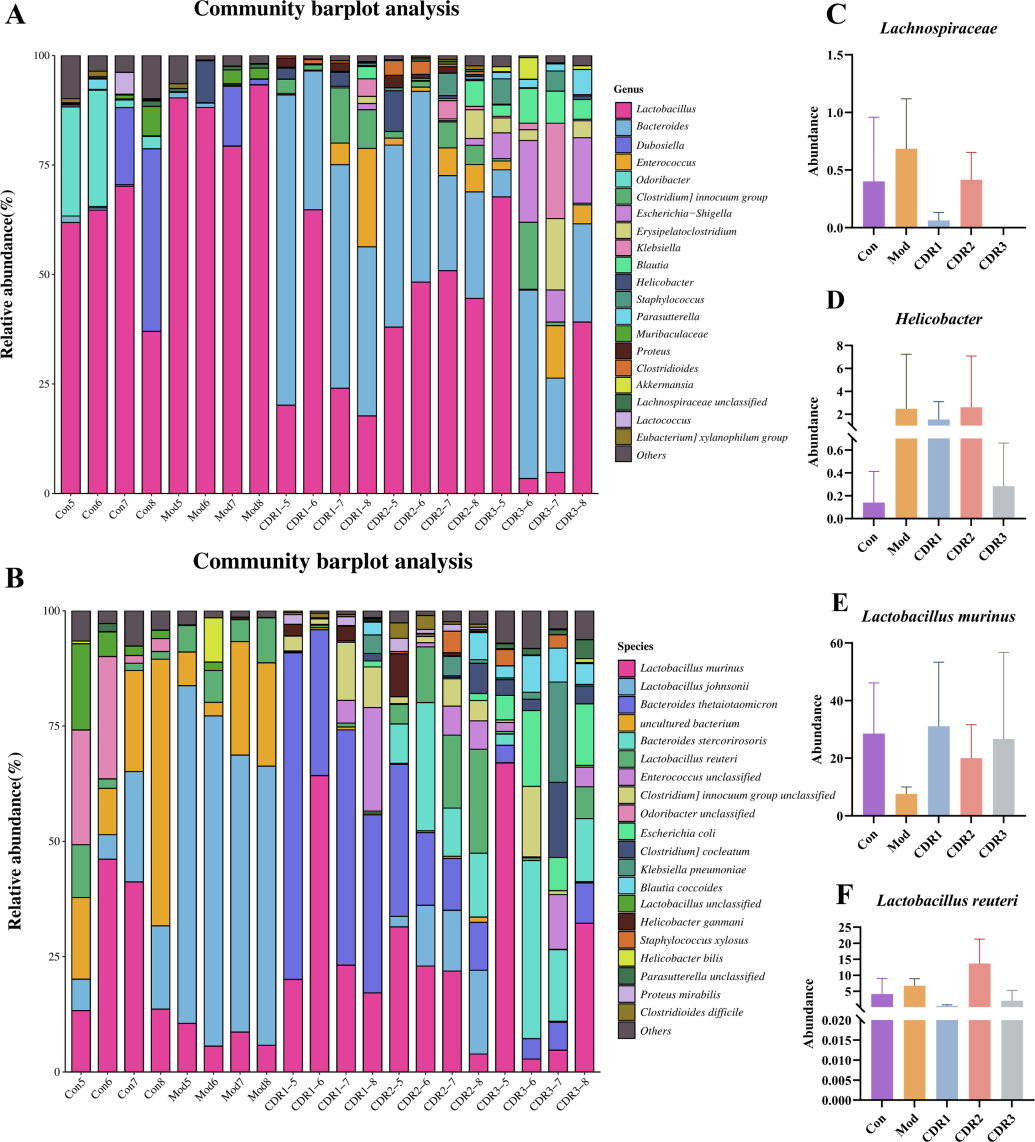
Genus-level and species-level community analysis of mice during the recovery period. **(A)** Relative abundance of the top 20 most abundant genera. **(B)** Relative abundance of the top 20 most abundant species; (C-F). Absolute abundance analysis: **(C)** Lachnospiraceae, **(D)** Helicobacter, **(E)** Lactobacillus murinus, **(F)** Lactobacillus reuteri. Data are presented as mean ± SD (n=4/group). Statistical significance was determined by one-way ANOVA followed by Tukey's post hoc test.

The composition of gut microbiota at the genus and species levels was also analyzed. At the genus level, *Lactobacillus* was the dominant genus in the Con, CDR1, CDR2, and CDR3 groups (**Figure 8A**). Systematic comparison revealed that CDR1 achieved the most effective restoration of key genera, including *Helicobacter* and *Lachnospiraceae*, with their relative abundances most closely matching those in the Con group (**Figures 8C, D**). At the species level, *Lactobacillus murinus* was the dominant species in all groups (**Figure 8B**). Comparative analysis demonstrated that CDR1 showed superior recovery of beneficial species, including *Lactobacillus reuteri* and *L. murinus*, with abundances significantly closer to normal levels compared to other treatment groups (**Figures 8E, F**). These results indicate that the CDR1 and CDR3 could effectively ameliorate gut microbiota dysbiosis after recovery.

Phenomenally, the restoration of host-microbiota homeostasis upon CDR1 treatment was evidenced by prompt microbial reconstitution after therapy, including probiotic and pathogenic normalization, beneficial consortia stabilization (e.g., *Bacteroides thetaiotaomicron* and *L. murinus*), together with a convergent tendency of 15 differential species revealed in LEfSe analysis toward the Con group (**Supplementary Figure S4**). Functional investigation also revealed that increased metabolic activity and DNA repair explained this stability of the microbiota (**Supplementary Figure S3**).

Taken together, these results demonstrate that the CDR1 treatment provides comprehensive advantages over other therapeutic approaches, not only most effectively improving gut microbiota dysbiosis during active treatment but also maintaining ecological balance after treatment cessation. This provides substantial evidence for the long-term modulatory effects of CDR1 on the gut microbiome.

### 3.5 Conventional combination reduces the risk of CDI recurrence

Because the restoration of colonization resistance is critical for preventing CDI recurrence (Lawley and Walker, 2013; Perez-Cobas et al., 2015), we next performed a *C. difficile* spore re-challenge to assess the long-term protective efficacy of each treatment. Upon re-challenge, the CDR1-treated mice demonstrated the most robust colonization resistance. Fecal *C. difficile* load progressively decreased in this cohort and was accompanied by a lack of further superinfection (**Figures 2A–C**). The other combination regimens, CDR2 and CDR3-treated mice, also did not develop severe relapse but showed weaker protection with transient or delayed control of bacterial loads. Conversely, the two monotherapies each yielded a recurrence. The Van group experienced a transient clinical deterioration and bacterial resurgence. The SXD group also experienced a bacterial rebound over time, with the final load exceeding its initial level (**Figure 2A**).

When re-challenged, an analysis of the colonic tissue further showed chronic effects on mucosal integrity associated with each regimen. The suppression in mucosal destruction paralleled the degree of this process that was present in Van monotherapy-induced disease, which exhibited extreme mucosal disruption with extensive shedding of epithelial cells. Groups CDR2 and CDR3 showed partial inflammatory infiltration or loss of epithelial cells. Notably, the CDR1 and SXD groups displayed the most effective mucosal protection, with the least tissue damage and inflammation observed among all groups (**Figure 2D**).

Together, these data show that the CDR1 regimen is the most effective way to prevent CDI recurrence long term by completely restoring colonization resistance and maintaining intact intestinal mucosal functions.

### 3.6 Effects of different combination regimens on gut microbiota at distinct treatment stages

Assessment and comparison of microbiota composition after treatment indicated parallel but distinct outcomes between three regimens (CDR1, CDR2, CDR3). LEfSe analysis indicated that the low-dose schedule (CDR3) significantly enriched *Parabacteroides goldsteinii* as compared with CDR1, whereas a greater abundance of Firmicutes and Lactobacillales was present in CDR1 than in CDR2(**Supplementary Figures S5A, B**, **S6A, B**). The two regimens also differed in their predicted metabolic landscape, with CDR1 being more enriched for pathways associated with Firmicutes colonization, and CDR2 being more abundant in oxidative phosphorylation (**Supplementary Figure S5C**). CDR3 also had a higher abundance of the flagellar assembly protein, FlgJ, compared with CDR1, which may indicate an increase in colonization-associated taxa (**Supplementary Figure S6C**).

And, even in the recovery phase, the composition of microbiota was significantly different between regimens. CDR1 was characterized by higher *Bacteroides thetaiotaomicron* relative abundance compared to CDR2 and CDR3. CDR2 (short-course vancomycin) was enriched in *Lactobacillus johnsonii* and *Lactobacillus reuteri* compared with CDR1, while CDR3 (low-dose vancomycin) showed marked expansion of potentially pathogenic taxa such as *Escherichia coli* and *Escherichia–Shigella* (**Supplementary Figures S7A, B**, **S8A, B**). LEfSe analysis indicated that CDR2 and CDR1 were more similar in microbial structure, with fewer discriminatory taxa between them, whereas CDR3 differed substantially from both. The predicted metabolic profiles during the recovery phase also differed between the two treatment regimens. PICRUSt2-derived KEGG pathway prediction combined with STAMP analysis further demonstrated that the lipid metabolism pathways were at higher enrichment in CDR2 and CDR3 groups, while secondary metabolite biosynthesis was more abundant for predicted function comparisons between CDR1 and the other two samples. These findings suggest that different medication strategies may influence therapeutic outcomes by modulating nutrient metabolism patterns (**Supplementary Figure S7C**, **S8C**).

Overall, these findings indicate that the antibiotic dosing strategy significantly influences gut microbiota remodeling. CDR1 most completely returned to a balanced community, while CDR2 retained major probiotic groups but with partial similarity, and CDR3, although beneficial in some aspects, was also enriched with potential pathogens, indicating the need for additional improvement.

## 4 Discussion

The clinical management of CDI is profoundly challenged by its high recurrence rates and treatment resistance. To address the high recurrence rate in CDI clinical management and the potential negative impact of vancomycin on gut microecology (Isaac et al., 2017; Aslam et al., 2005), this study systematically designed and compared vancomycin monotherapy with three combination regimens of vancomycin and Shengjiang Xiexin Decoction (SXD). The treatment protocol, referencing the American College of Gastroenterology (ACG) 2021 Clinical Guidelines (Kelly et al., 2022), consisted of a 10-day treatment period and an equivalent recovery period. The vancomycin dose was determined based on multiple clinical studies (Fitzpatrick et al., 2012; Jose et al., 2021; Lv et al., 2015; Tsai et al., 2022), ultimately adopting 50 mg/kg/day as the mouse equivalent dose. This dose was derived from the standard adult regimen (125 mg, four times daily) converted via body surface area, ensuring intraluminal drug concentrations exceed the *C. difficile* minimum inhibitory concentration while minimizing systemic exposure risk. In the combination therapy design, besides the standard combination regimen (CDR1), the CDR2 group was established to evaluate a phased drug withdrawal strategy, and the CDR3 group was designed to simulate tapered/pulsed dosing regimens for recurrent CDI (Sirbu et al., 2017), aiming to explore the clinical feasibility of reducing overall antibiotic exposure.

Efficacy evaluation revealed that the CDR1 regimen performed best across comprehensive indicators. Compared to CDR2, CDR3, and monotherapy groups, CDR1 exerted its therapeutic effects in stages: during the acute treatment phase, it rapidly controlled the *C. difficile* load, creating a window of low pathogen burden for subsequent microbiota repair; during the recovery phase, it significantly promoted the restoration of gut microbiota structure and the establishment of colonization resistance, specifically manifested as a significant increase in gut microbiota α-diversity (Shannon, Simpson indices), effective restoration of key beneficial bacteria such as *Bacteroides thetaiotaomicron* and *Akkermansia muciniphila*, and demonstrated stronger anti-colonization capability upon recurrence challenge. In contrast, CDR2 and CDR3, due to insufficient vancomycin dosage or interrupted treatment course, resulted in incomplete initial pathogen suppression, potentially leading to early recurrence, thereby undermining the synergistic foundation of SXD.

Further analysis indicated that CDR1 specifically promoted the recovery of *B. thetaiotaomicron* and *A. muciniphila*, two bacterial species with mucosal protective functions. Studies show that *A. muciniphila* not only possesses anti-inflammatory properties but also enhances intestinal barrier function (Grander et al., 2020); in a mucin environment, it can induce macrophage polarization towards an anti-inflammatory phenotype, with high expression of IL-10, Arg1, and low expression of pro-inflammatory factors like IL-1β, IL-6 (Kim et al., 2023). *B. thetaiotaomicron* can strengthen epithelial defense by regulating mucosa-associated gene expression and alleviate complement activation-induced mucosal damage (Bäckhed et al., 2005). We speculate that SXD may act as a prebiotic or metabolic modulator, supporting the colonization and function of these strains. Its metabolite short-chain fatty acids not only provide energy for intestinal epithelial cells (Dao et al., 2016) but also promote Lgr5+ intestinal stem cell proliferation and goblet cell differentiation, thereby thickening the mucus layer and enhancing barrier integrity (Kim et al., 2021), establishing a physical defense line against pathogens.

Beyond barrier repair, we propose that CDR1’s synergistic mechanism also involves a “metabolic inhibition pathway,” directly suppressing *C. difficile* recurrence by modulating bile acid metabolism. Existing studies confirm that primary bile acids (e.g., CA) promote spore germination, whereas secondary bile acids (e.g., DCA, LCA) inhibit *C. difficile* growth (Buffie et al., 2014). While vancomycin effectively clears vegetative cells, it also inhibits secondary bile acid-producing bacteria, leading to an imbalance in the bile acid profile and creating a “permissive environment” for spore germination (Theriot et al., 2014). Active components in SXD (e.g., baicalin, berberine) have been reported to modulate microbiome function and influence bile acid metabolic enzyme activity (Guo et al., 2016). Therefore, we hypothesize that after vancomycin clears the pathogen, SXD, by remodeling the microbiota, promotes the generation of secondary bile acids, blocking spore germination and disease recurrence at the metabolic level.

In summary, the success of the CDR1 regimen stems from the synergy and complementarity between vancomycin and SXD: vancomycin provides initial pathogen control, while SXD drives microbiota and metabolic reconstruction, shifting the treatment strategy from mere pathogen clearance to holistic ecological restoration.

Despite these promising findings, this study has several noteworthy limitations. Firstly, although considerable effort was made to simulate clinical practice in *C. difficile* strain selection (using strain 027) and treatment regimen design, the findings derived from this murine model inherently require further validation through rigorous clinical trials to ascertain the true therapeutic potential and safety of these combination regimens in human subjects. Secondly, while key inflammatory markers and barrier proteins were assessed, our analysis of the synergistic mechanisms between SXD and vancomycin remains primarily at the microbiota and metabolic levels; its impact on bile acid receptor signaling pathways (e.g., FXR, TGR5) remains unclarified and requires further exploration combining metabolomics and molecular biology techniques. Furthermore, fecal *C. difficile* qPCR detection cannot distinguish between viable bacteria, dead bacteria, and spores; subsequent studies are recommended to incorporate toxin detection or transcriptomic analysis for more accurate efficacy assessment. Additionally, as a complex traditional Chinese medicine formula, SXD requires enhanced chemical standardization research in the future to improve result reproducibility. Particular attention must be paid to the potential safety risks of SXD. Although no significant adverse reactions were observed in this study, literature reports indicate that its constituent herb Pinellia ternata (raw Banxia) can cause mucosal irritation (Fueki et al., 2020) and neurotoxicity (Zou et al., 2023), and the main component of Coptis chinensis (Huanglian), berberine, may cause gastrointestinal discomfort (Tondt and Bays, 2022) and rare hepatocardiac adverse reactions (Zhang et al., 2018) at high doses or with long-term use. The safety observed in this study might be attributed to the standardized processing of Pinellia, the detoxifying effects of formula compatibility, and short-term administration at clinically equivalent doses. However, the limitations of animal experiments necessitate the establishment of a systematic drug safety monitoring system in future clinical studies.

Finally, this study could not fully mimic the complex clinical characteristics frequently observed in human CDI patients, including common comorbidities such as malnutrition, various degrees of immune compromise, and polypharmacy. The relatively uniform and healthy baseline status of our experimental animals starkly contrasts with the prevalent multi-morbidity in clinical patients. This significant discrepancy poses challenges for the direct translational application of our research findings into diverse clinical practice settings. These limitations collectively underscore the imperative for future research to develop animal models that more closely approximate complex clinical scenarios and to conduct robust, multi-center clinical validations.

The findings of this study not only provide new ideas for the clinical treatment of CDI but also reveal the broad prospects of combining antibiotics with traditional Chinese medicine. In the context of the increasingly severe global health challenge of antibiotic resistance, traditional Chinese medicine formulas like SXD demonstrate their unique value: on one hand, they can act as “microecological restorative agents for antibiotic-induced damage,” effectively mitigating the impact of broad-spectrum antibiotics on gut microecology; on the other hand, by restoring the host’s own defense capabilities rather than directly killing pathogens, they offer a potential strategy for “reducing the development of antibiotic resistance.” Although this study did not directly verify its impact on resistance, treatment strategies based on microbiota reconstruction and metabolic regulation are expected to reduce reliance on antibiotics alone, thereby playing a significant role in the management of a broader range of infectious diseases.

## 5 Conclusion

This study highlights the differential strengths of monotherapies and the advantages of integrated treatment strategies for CDI. While vancomycin monotherapy provides rapid infection control and SXD monotherapy helps preserve the intestinal barrier to reduce relapse risk, their combination produces a synergistic effect, addressing both acute infection management and long-term outcome improvement. In particular, the combination of SXD and vancomycin produces a synergy effect on early amelioration of symptoms as well as a long-term protective effect against recurrence. Significantly, vancomycin dosing regimen stood out as a key factor in long-term outcomes. With regard to the CDR1, a better performance was observed in to prevention of recurrent CDI and promoting recovery of stable gut microbial communities. In comparison, de-escalation schedules (CDR2 and CDR3), although effective in curing acute symptoms, were associated with a higher relapse rate and altered microbiota homeostasis. These results indicate that potentially reducing antibiotic exposure could inadvertently compromise long-term treatment efficacy. In summary, this study emphasizes the importance of retaining recommended-dose vancomycin in concomitant regimens with SXD for optimal results. This balance between short-term effectiveness and durable relapse prevention allows to underscores the need for an appropriate antibiotic stewardship in the treatment of CDI.

## Data Availability

The original contributions presented in the study are included in the article/**Supplementary Material**. Further inquiries can be directed to the corresponding authors.
