# Supplementary material for: Shengjiang Xiexin decoction combined with vancomycin for Clostridioides difficile infection: impact of vancomycin dose-reduction strategy on gut microbiota homeostasis and recurrence risk
Source: Front Cell Infect Microbiol. 2026 Feb 11;15:1740179. doi: 10.3389/fcimb.2025.1740179 (PMC12932506; doi:10.3389/fcimb.2025.1740179)
Supplement: Supplementary file 1 [file DataSheet1.docx]

Supplementary Material

**Shengjiang Xiexin decoction Combined with Vancomycin for *Clostridioides difficile* Infection: Impact of Vancomycin Dose-Reduction Strategy on Gut Microbiota Homeostasis and Recurrence Risk**

Lin Zhu^1^^#^, Li-sheng Chen^1#^, Fu-zhi Ma^1^, Jin-e Peng^1^, Yu-bu Wang^1^, Yu-qing Ma^1^, Yue Xu^1^, Yi Wang^1^, Ayiman Yeerjiang^1^, Cong-en Zhang^2^*, Zhi-jie Ma^1^*

^1^ Department of Pharmacy, Beijing Ditan Hospital, Capital Medical University, 100015, Beijing, China

^2^ Department of Pharmacy, Beijing Friendship Hospital, Capital Medical University, 100050, Beijing, China

^#^**These authors are co-first authors of the article**

***Correspondence:**Zhi-jie Ma, Beijing Ditan Hospital, Capital Medical University, 8 Jingshun East Street, 100015, Beijing, China. E-mail: mazj2021@163.com.

Cong-en Zhang, Beijing Friendship Hospital, Capital Medical University, No. 95, Yongan Road, 100050, Beijing, China. E-mail: [zce820@163.com](mailto:zce820@163.com).

# Microbiota Dynamics During CDR1 Treatment

A focused analysis of the CDR1 regimen highlighted its robust restorative effects. Heatmap and LEfSe analyses revealed that the abundance of key species, such as Lactobacillus murinus, and the overall community structure in the CDR1 group approached that of the Con group (Supplementary Figure 1A, B, D, F; Supplementary Figure 2). Circos analysis confirmed that the relative abundance of key functional bacteria (e.g., *Lactobacillus murinus*) in the treatment group was close to that in the Con group (Supplementary Figure 1 C, E). Functional prediction also showed enrichment of immune-related pathways in the CDR1 group (Supplementary Figure 1G).

**
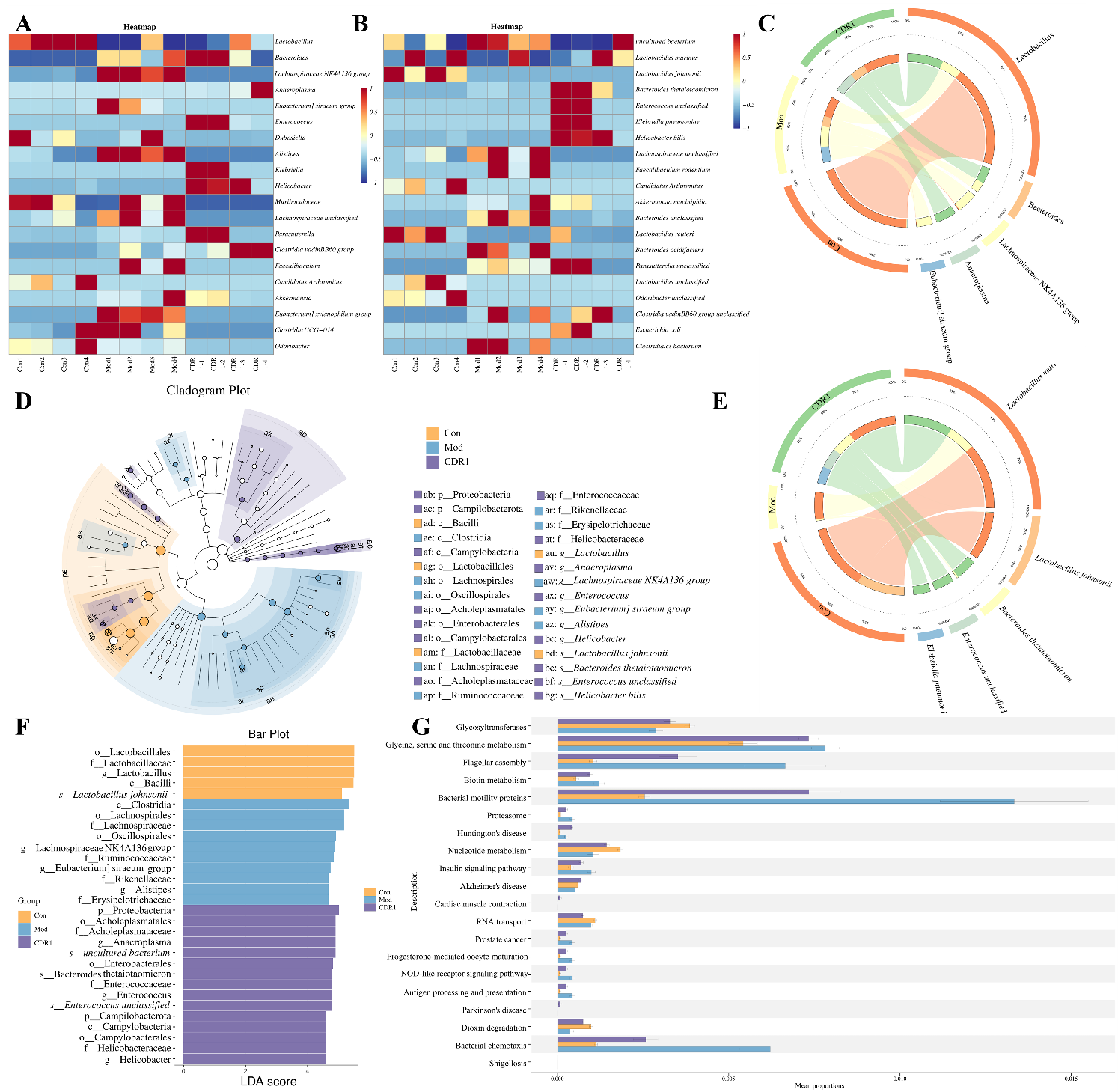
**

**Supplementary Figure 1.** **Microbiota analysis of the conventional treatment regimen (CDR1) during the treatment phase.** (A) Genus-level heatmap; (B) Species-level heatmap; (C) Genus-level abundance Circos plot; (D) LEfSe cladogram plot; (E) Species-level Circos plot; (F) LEfSe bar plot; (G) Differential microbial metabolic pathways based on PICRUSt2 prediction and STAMP analysis. Data are presented as mean ± SD, n = 4 per group.

# CDR1 Treatment-Induced Shifts in Bacterial Abundance

We conducted an in-depth analysis of the differential microbiota identified by LEfSe analysis after the treatment phase. The results revealed that all 15 bacterial species showing significant differences during the treatment phase exhibited a trend of converging toward the control (Con) group during the recovery phase. Boxplot visualization clearly demonstrated that the abundances of these 15 differential species were restored to varying degrees following the CDR1 combination therapy. Notably, the abundances of beneficial bacteria such as *Lactobacillus* and *Bacteroides thetaiotaomicron* were significantly increased, indicating a favorable recovery pattern. These findings suggest that the CDR1 treatment regimen effectively promotes the restoration of gut microbiota structure and abundance in mice, contributing to the reestablishment of a healthy microbial community.


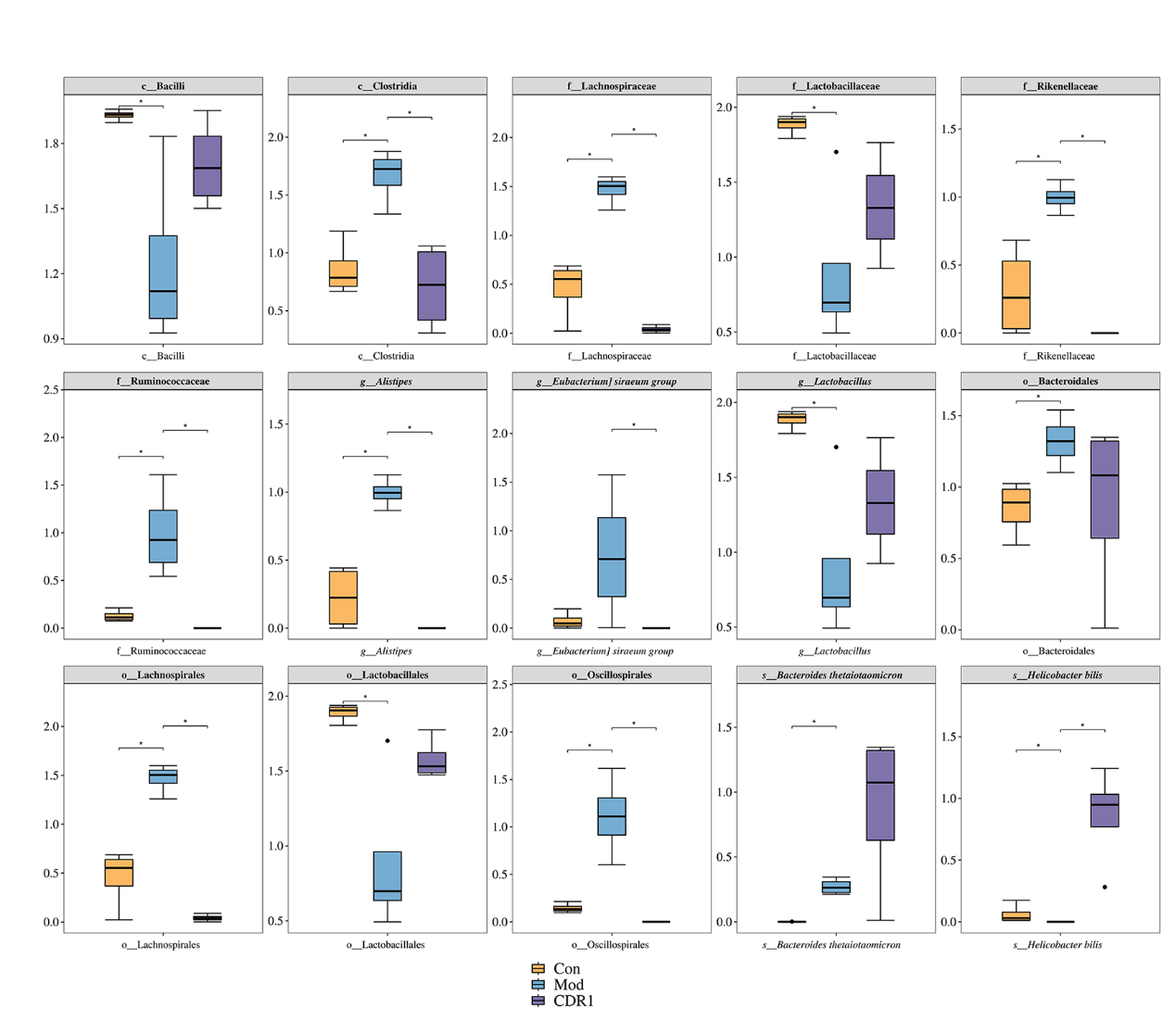


**Supplementary Figure 2. Differentially abundant taxa identified by LEFSe analysis during the treatment phase.**

# Gut Microbiota Recovery Dynamics Following CDR1 Treatment

Further analysis of CDR1's effect on gut microbiota during recovery revealed that after discontinuing treatment, probiotics (e.g., *Lactobacillus johnsonii*) and potential pathogens (e.g., *Helicobacter*) in the CDR1 group approached normal levels (Supplementary Figure. 3A, B). Furthermore, *Bacteroides thetaiotaomicron* and *L. murinus* formed a stable dominant microbiota, maintaining ecological balance through sustained proliferation (Supplementary Figure. 3C, E). LEfSe analysis revealed distinct microbial biomarkers between experimental groups during the recovery phase (Supplementary Figure 3D, F). KEGG pathway enrichment analysis (Supplementary Figure. 3G) revealed that CDR1 maintains long-term microbiota stability by regulating microbial metabolic networks and DNA repair systems.

**
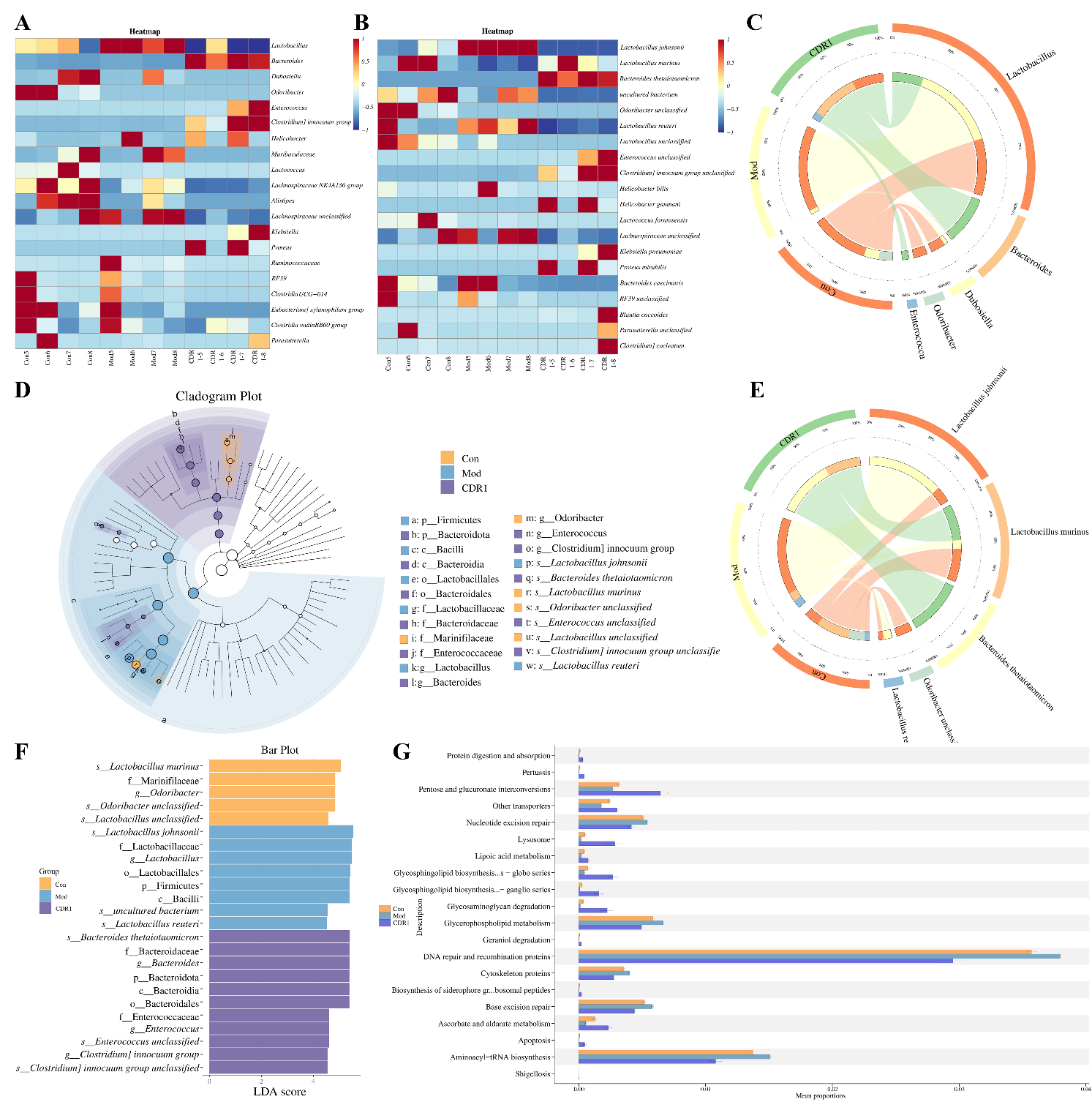
**

**Supplementary Figure 3. Microbiota analysis of the conventional treatment regimen (CDR1) during the recovery phase.** (A) Genus-level heatmap; (B) Species-level heatmap; (C) Genus-level abundance Circos plot; (D) LEfSe cladogram plot; (E) Species-level Circos plot; (F) LEfSe bar plot; (G) Differential microbial metabolic pathways based on PICRUSt2 prediction and STAMP analysis. Data are presented as mean ± SD, n = 4 per group.

# Post-CDR1 Treatment Bacterial Abundance Recovery Dynamics

We conducted an in-depth analysis of the recovery dynamics of differential microbiota identified by LEfSe analysis after the conclusion of treatment. The results revealed that five bacterial taxa (c_Bacilli, f_Lactobacillaceae, *g_Lactobacillus*, o_Bacteroidales, o_Lactobacillales), which had already shown a recovery trend during the treatment phase, exhibited further abundance shifts toward the level of the control (Con) group after CDR1 treatment was discontinued. Boxplot analysis further confirmed that 15 bacterial taxa among those identified by LEfSe showed varying degrees of abundance recovery during the recovery phase. These findings indicate that CDR1 not only effectively modulates the microbiota during the treatment period but also continues to promote the restoration of gut microbial structure and ecological balance after treatment cessation.


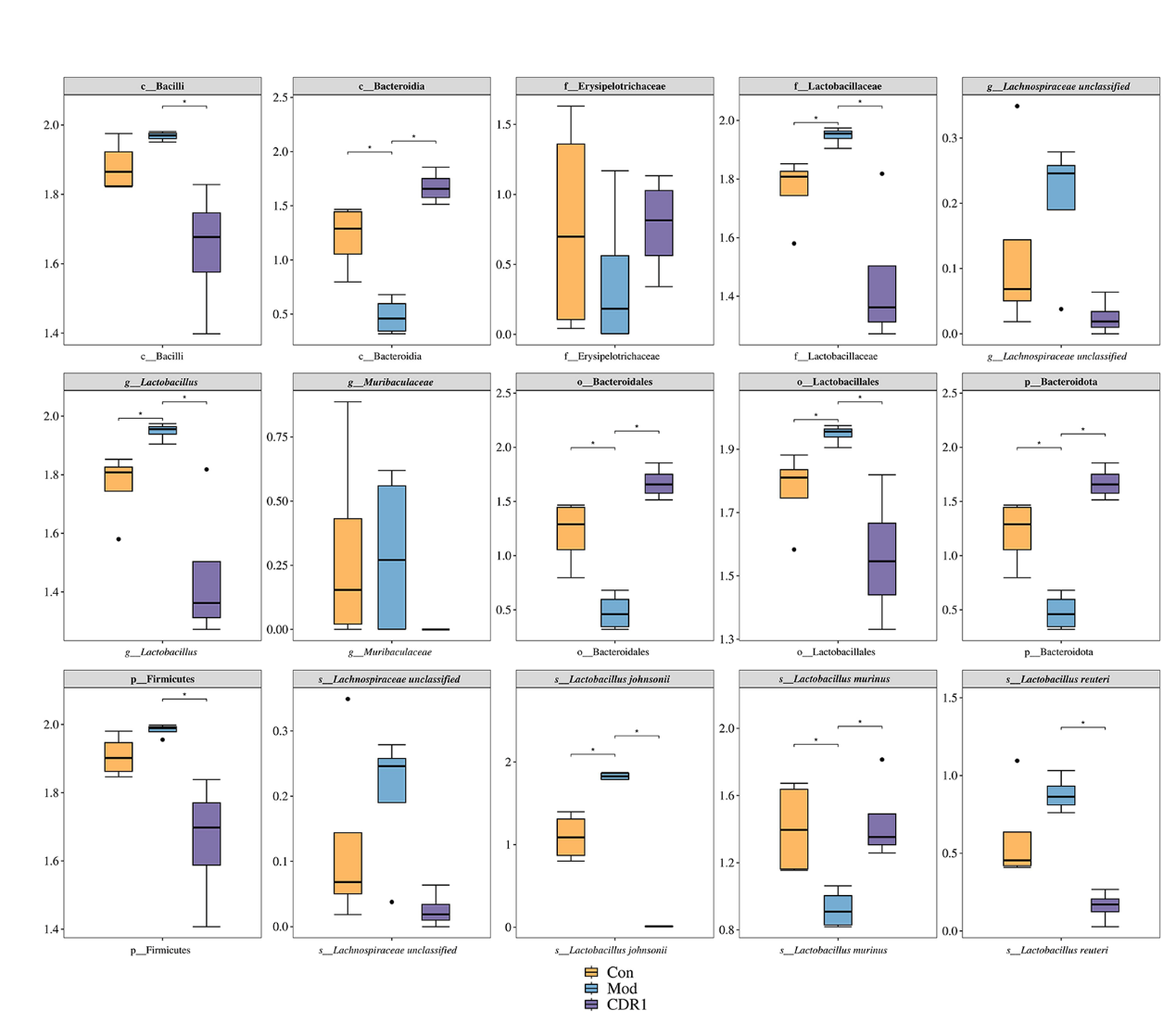


**Supplementary Figure 4. Differentially abundant taxa identified by LEFSe analysis during the recovery phase.**

# Standard Combination Regimen (CDR1) vs. Short-Course (CDR2) and Reduced-Dose (CDR3) Vancomycin: Staged Effects on Intestinal Microbiota

## Comparative Analysis of Gut Microbiota Modulation Between CDR1 and CDR2 Regimens During the Treatment Phase

Given that gut microbiota dysbiosis is a key aspect to be managed in the treatment of *Clostridioides difficile* infection (CDI), this study comparatively analyzed the dynamic changes in the gut microbiota of mice under different intervention regimens during the treatment period. The results showed that, during the treatment phase, different therapeutic regimens had significantly divergent effects on the structure and function of the gut microbiota. Notably, in the combination therapy group, the full-dose full-course regimen (CDR1) exhibited a significant increase in the relative abundance of Firmicutes and Lactobacillales during treatment. Firmicutes include multiple butyrate-producing species, and butyrate has been demonstrated to play important roles in ameliorating intestinal inflammation, enhancing barrier function, and preventing bacterial translocation (Tian et al., 2023). Furthermore, previous studies have indicated that probiotics formulated with strains from the order Lactobacillales can reduce the incidence of primary CDI (Maziade et al., 2015). Together, these findings suggest that the standard full-course vancomycin treatment may exert a positive influence on the gut microecology by promoting the colonization or proliferation of these beneficial microbial communities. (Supplementary Figure 5A, B).

Functional prediction analysis revealed significant differences in metabolic pathways between the CDR1 and CDR2 groups, with the phosphotransferase system (PTS) and oxidative phosphorylation pathways showing the most notable variations. The CDR1 group exhibited a marked increase in PTS pathway abundance, which may promote the colonization advantage of Firmicutes through carbon catabolite repression (Xu et al., 2023), thereby exerting therapeutic effects by competitively inhibiting pathogen colonization (Drissi et al., 2015). In contrast, the CDR2 group demonstrated significantly higher activity in the oxidative phosphorylation pathway. As a core pathway for energy metabolism in intestinal epithelial cells, dysfunction of oxidative phosphorylation may lead to lipid accumulation and impaired barrier function (Moschandrea et al., 2024), suggesting that CDR2 likely improves intestinal barrier integrity primarily by modulating host energy metabolism. In summary, we propose that CDR1 and CDR2 exert their therapeutic effects through distinct metabolic mechanisms (Supplementary Figure 5C).

**
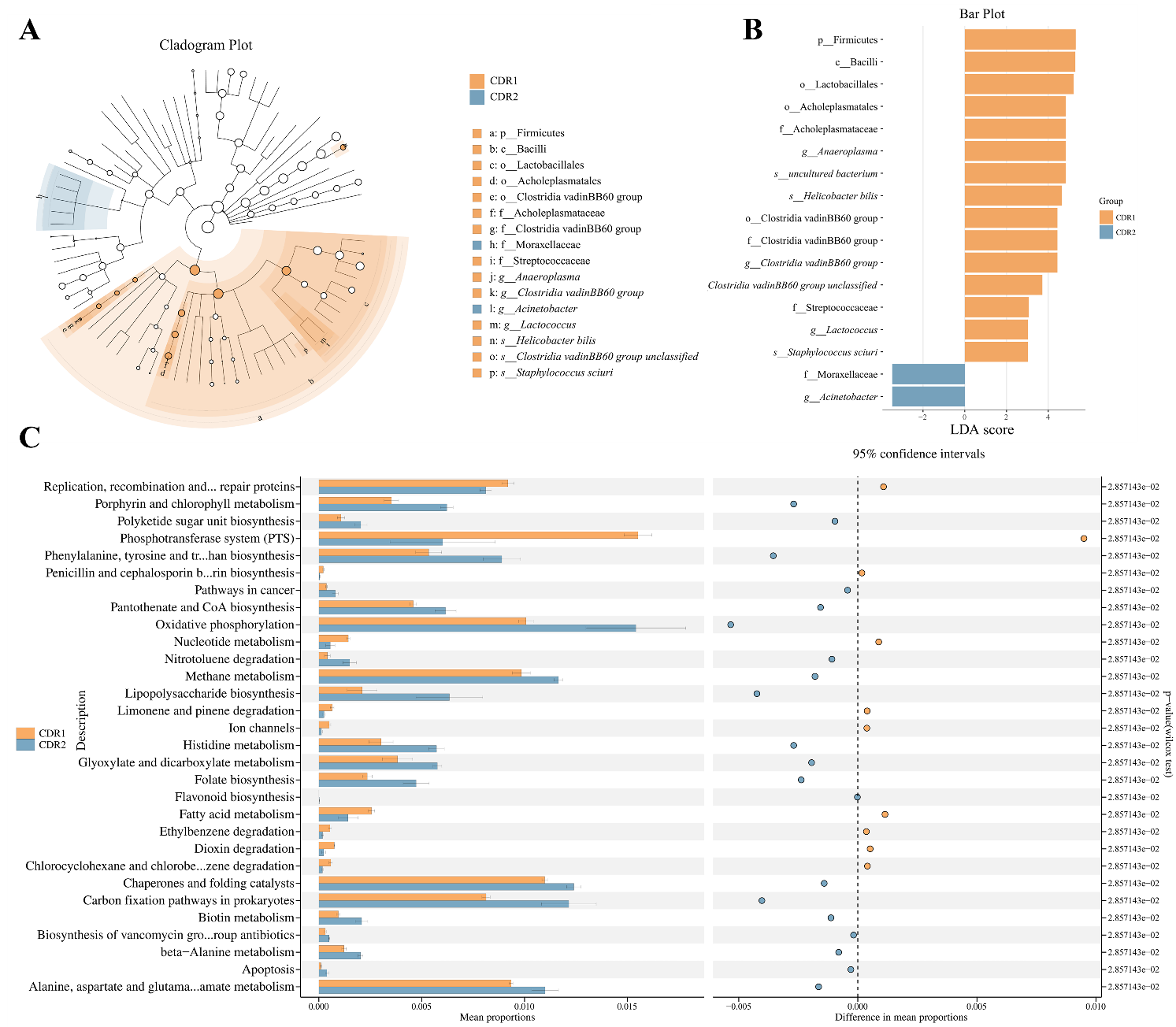
**

**Supplementary Figure 5. Microbiota analysis of the Conventional Combined group (CDR1) versus the Short-Course Combined group (CDR2) during the treatment phase.** (A) LEfSe cladogram plot, (B) LEfSe bar plot, (C) Differential microbial metabolic pathways based on PICRUSt2 prediction and STAMP analysis.

## Comparative Analysis of Gut Microbiota Modulation Between CDR1 and CDR3 Regimens During the Treatment Phase

Meanwhile, we further investigated the effects of reducing the vancomycin dosage in the combination therapy on the gut microbiota of mice. LEfSe analysis revealed that the abundance of *Parabacteroides goldsteinii* was significantly higher in the CDR3 group (with reduced vancomycin dosage) compared to the CDR1 group (Supplementary Figure 6A,B). Previous studies have demonstrated that *Parabacteroides goldsteinii* can alleviate symptoms of DSS-induced colitis (Gerkins et al., 2022) and drug-induced intestinal injury and barrier disruption (Li et al., 2024), suggesting that the CDR3 regimen may improve the intestinal environment by promoting the enrichment of this gut barrier-protective bacterium.

Through STAMP analysis of predicted pathways, differential pathway screening identified that the abundance of the flagellar assembly protein FlgJ was significantly elevated in the CDR3 group compared to the CDR1 group (Supplementary Figure 6C). Given that colonization by *Clostridioides difficile* is a prerequisite for its pathogenicity, and considering the critical role of FlgJ in bacterial flagellar assembly (Zhang et al., 2012) coupled with the dependence of *C. difficile* pathogenicity on flagella-mediated colonization capacity (Sidner et al., 2023), this finding implies that reducing the vancomycin dosage may increase the risk of pathogen colonization.

**
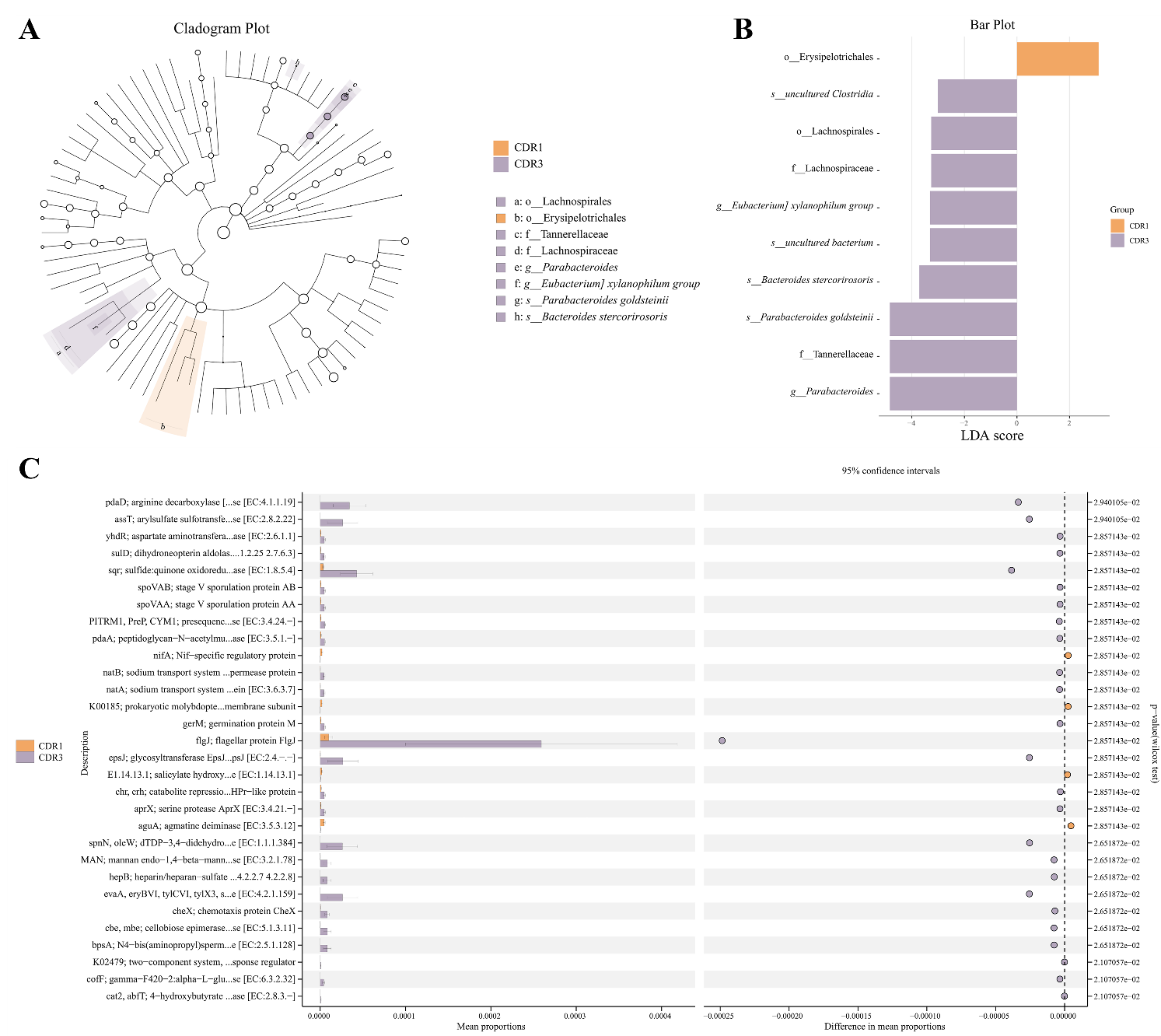
**

**Supplementary Figure 6. Microbiota analysis of the Conventional Combined group (CDR1) versus the Low-dose combination (CDR3) during the treatment phase.** (A) LEfSe cladogram plot, (B) LEfSe bar plot, (C) Differential microbial metabolic pathways based on PICRUSt2 prediction and STAMP analysis.

## Comparative Analysis of Gut Microbiota Modulation Between CDR1 and CDR2 Regimens During the Recovery Phase

Through an in-depth analysis of the gut microbiota in patients during the recovery phase under different vancomycin treatment regimens, this study explored the significant impact of antibiotic dosing strategies on the reconstruction of gut microecology. Notably, distinct reduction strategies exerted markedly different effects on microbiota recovery. The CDR2 group, characterized by a shortened duration of vancomycin use, exhibited a typical enrichment pattern of probiotic bacteria, with significantly increased relative abundances of *Lactobacillus johnsonii* and *Lactobacillus reuteri* compared to the CDR1 group (Supplementary Figure 7A, B). These two *lactobacilli* species enhance intestinal epithelial barrier function through mechanisms such as maintaining tight junction integrity and competitively inhibiting pathogen adhesion (Liu et al., 2015).

Further KEGG pathway prediction based on PICRUSt2 and subsequent STAMP analysis revealed that the predicted gene abundances for pathways related to plant-derived secondary metabolites and lipid metabolism—including Flavone and flavonol biosynthesis, Biosynthesis of unsaturated fatty acids, and Glycerophospholipid metabolism—were significantly lower in the CDR1 group than in the CDR2 group(Supplementary Figure 7C). A reduction in the Flavone and flavonol biosynthesis pathway abundance may lead to increased oxidative stress (Chen et al., 2024), while a decline in the Biosynthesis of unsaturated fatty acids pathway—which helps maintain intestinal barrier function—could contribute to impaired gut barrier integrity (Pothoulakis 2000). These functional differences are highly consistent with the previously observed changes in pharmacological efficacy indicators, providing molecular-level evidence that different medication strategies alter the metabolic patterns of nutrients.

**
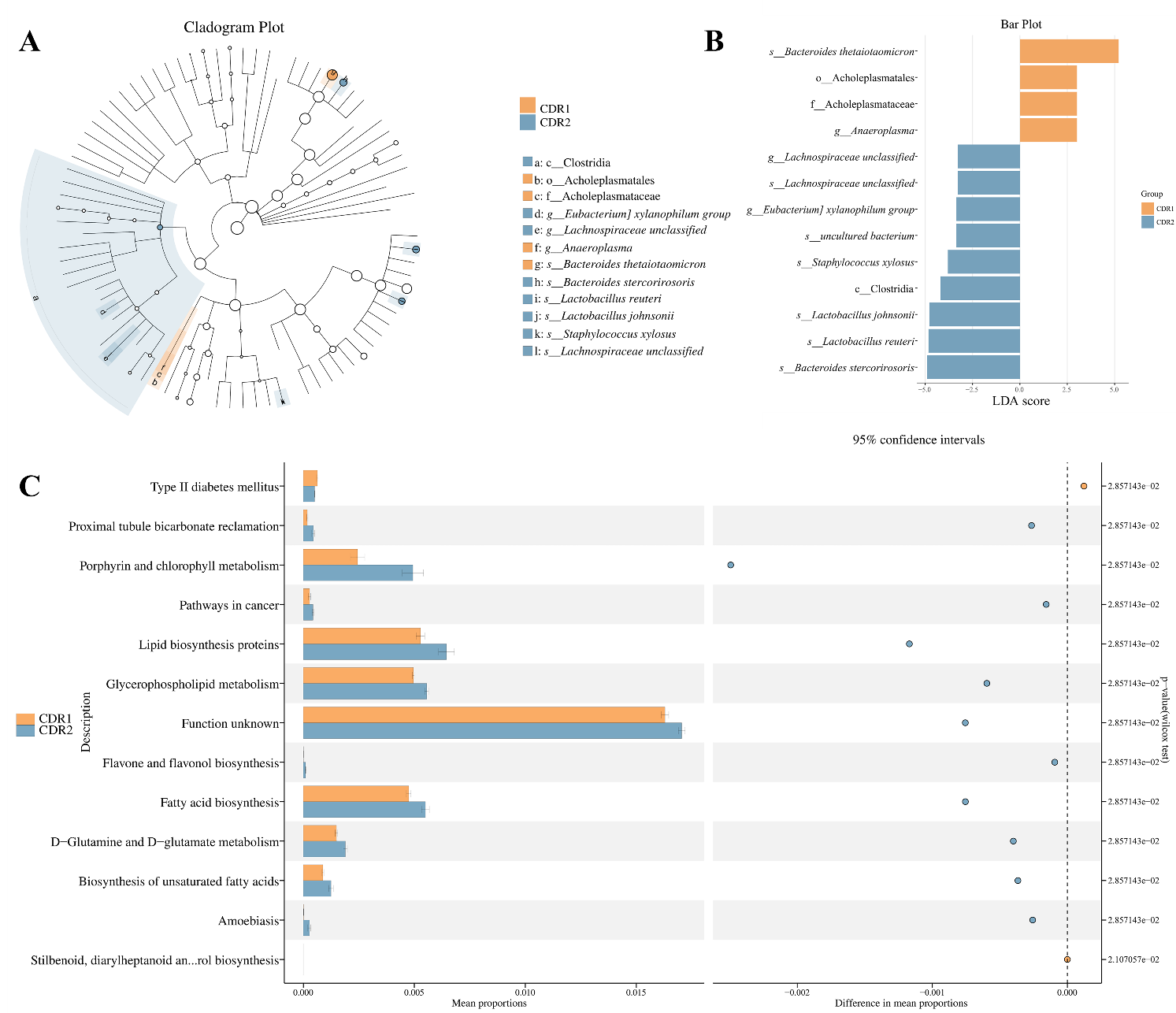
**

**Supplementary Figure 7. Microbiota analysis of the Conventional Combined group (CDR1) versus the Short-Course Combined group (CDR2) during the** **recovery phase.** (A) LEfSe cladogram plot, (B) LEfSe bar plot, (C) Differential microbial metabolic pathways based on PICRUSt2 prediction and STAMP analysis.

## Comparative Analysis of Gut Microbiota Modulation Between CDR1 and CDR3 Regimens During the Recovery Phase

In parallel, a comparative analysis was conducted between the CDR3 group, which received a low dose of vancomycin in the combination therapy, and the CDR1 group treated with the standard dose. LEfSe analysis (with an LDA threshold of 3) revealed significant differences in microbial composition among the three groups, primarily reflected in changes in the abundance of key functional bacteria. In the CDR1 group (standard vancomycin dose and course), the most characteristic dominant bacterium was *Bacteroides thetaiotaomicron*, whose relative abundance was significantly higher compared to the CDR3 group (Supplementary Figure 8A, B). This strain exhibits multiple probiotic functions: firstly, it significantly promotes the secretion of MUC2, the main component of the intestinal mucus layer, thereby enhancing physical barrier function; secondly, it regulates B cell differentiation to promote the production of secretory IgA, maintaining intestinal immune homeostasis(Wang et al., 2023); more importantly, this species has also been demonstrated to directly inhibit *Clostridioides difficile* (Hromada et al., 2021). These properties make it a crucial regulator of intestinal microecological balance.

Meanwhile, the microbial composition of the CDR3 group (low-dose regimen) showed potentially unfavorable alterations, characterized by a significant expansion of potentially pathogenic bacteria such as *Escherichia coli* and *Escherichia-Shigella*. These strains can exacerbate intestinal inflammatory responses by producing endotoxins and disrupting the epithelial barrier(Chi et al., 2021; Jia et al., 2025) (Supplementary Figure 8A, B). Further functional prediction analysis indicated that the abundance of pathways related to secondary metabolite biosynthesis—such as Stilbenoid, diarylheptanoid and gingerol biosynthesis—was significantly higher in the CDR1 group than in the CDR3 group (Supplementary Figure 8C). These findings further confirm that different medication strategies can influence the metabolic patterns of nutrients.

**
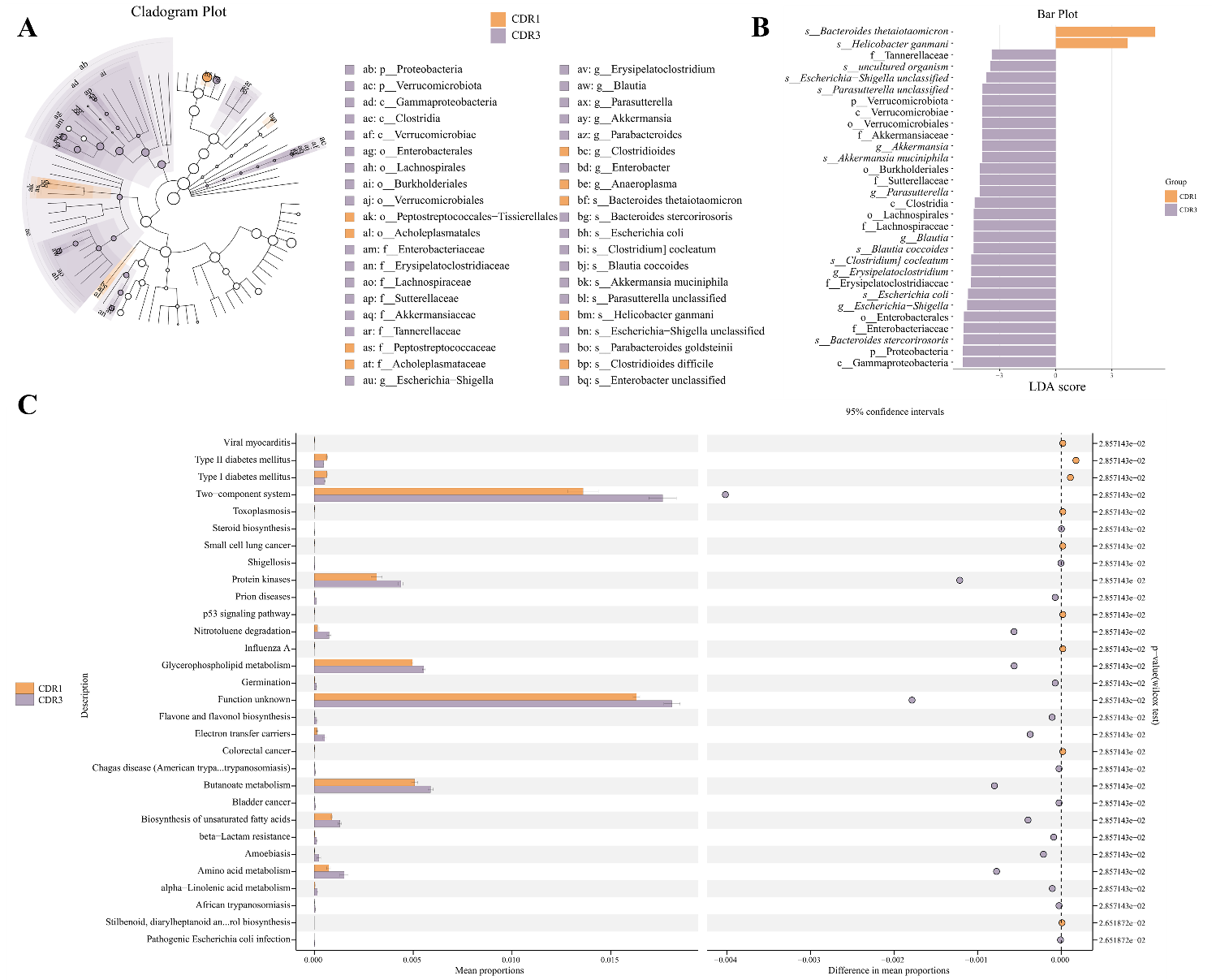
**

**Supplementary Figure 8. Microbiota analysis of the Conventional Combined group (CDR1) versus the Low-dose combination (CDR3) during the recovery phase.** (A) LEfSe cladogram plot, (B) LEfSe bar plot, (C) Differential microbial metabolic pathways based on PICRUSt2 prediction and STAMP analysis.

# References：

Tian, H., Cui, J., Ye, C., Zhao, J., Yang, B., Xu, Y., et al. (2023), Depletion of butyrate-producing microbes of the Firmicutes predicts nonresponse to FMT therapy in patients with recurrent *Clostridium difficile* infection. *Gut Microbes* 15, Article. doi: 10.1080/19490976.2023.2236362.

Maziade, P.-J., Pereira, P., Goldstein, E.J.C. (2015), A Decade of Experience in Primary Prevention of *Clostridium difficile* Infection at a Community Hospital Using the Probiotic Combination *Lactobacillus acidophilus* CL1285, *Lactobacillus casei* LBC80R, and *Lactobacillus rhamnosus* CLR2 (Bio-K+). *Clin. Infect. Dis.* 60, S144, Article. doi: 10.1093/cid/civ178.

Xu, T., Tao, X., He, H., Kempher, M.L., Zhang, S., Liu, X., et al. (2023), Functional and structural diversification of incomplete phosphotransferase system in cellulose-degrading clostridia. *Isme J* 17, 823, Article. doi: 10.1038/s41396-023-01392-2.

Drissi, F., Buffet, S., Raoult, D., Merhej, V. (2015), Common occurrence of antibacterial agents in human intestinal microbiota. *Front. Microbiol.* 6, Article. doi: 10.3389/fmicb.2015.00441.

Moschandrea, C., Kondylis, V., Evangelakos, I., Herholz, M., Schneider, F., Schmidt, C., et al. (2024), Mitochondrial dysfunction abrogates dietary lipid processing in enterocytes. *Nature* 625, 385, Article. doi: 10.1038/s41586-023-06857-0.

Gerkins, C., Oliero, M., Hajjar, R., Rendos, H.V., Fragoso, G., Calve, A., et al. (2022), THE MODULATION OF INTESTINAL INFLAMMATION BY PARABACTEROIDES GOLDSTEINII IN DEXTRAN SODIUM SULFATE INDUCED COLITIS IN MICE. *Gut* 71, A60, Meeting Abstract. doi: 10.1136/gutjnl-2022-IDDF.69.

Li, T., Ding, N., Guo, H., Hua, R., Lin, Z., Tian, H., et al. (2024), A gut microbiota-bile acid axis promotes intestinal homeostasis upon aspirin-mediated damage. *Cell Host & Microbe* 32, Article. doi: 10.1016/j.chom.2023.12.015.

Zhang, K., Tong, B.A., Liu, J., Li, C. (2012), A Single-Domain FlgJ Contributes to Flagellar Hook and Filament Formation in the Lyme Disease Spirochete *Borrelia burgdorferi*. *J. Bacteriol.* 194, 866, Article. doi: 10.1128/jb.06341-11.

Sidner, B., Lerma, A., Biswas, B., Do, T.V.T., Yu, Y., Ronish, L.A., et al. (2023), Flagellin is essential for initial attachment to mucosal surfaces by *Clostridioides difficile*. *Microbiol Spectr* 11, Article. doi: 10.1128/spectrum.02120-23.

Liu, H.-Y., Roos, S., Jonsson, H., Ahl, D., Dicksved, J., Lindberg, J.E., et al. (2015), Effects of *Lactobacillus johnsonii* and *Lactobacillus reuteri* on gut barrier function and heat shock proteins in intestinal porcine epithelial cells. *PHYSIOL REP* 3. doi: 10.14814/phy2.12355.

Chen, Z., Zhang, L., Lv, Y., Qu, S., Liu, W., Wang, K., et al. (2024), A genome assembly of ginger (*Zingiber officinale* Roscoe) provides insights into genome evolution and 6-gingerol biosynthesis. *Plant J.* 118, 682, Article. doi: 10.1111/tpj.16625.

Pothoulakis, C. (2000), Effects of *Clostridium difficile* toxins on epithelial cell barrier. *Ann. N. Y. Acad. Sci.* 915, 347, ; Review.

Wang, X.Y., Lin, S.S., Wang, L., Cao, Z.P., Zhang, M.M., Zhang, Y.F., et al. (2023), Versatility of bacterial outer membrane vesicles in regulating intestinal homeostasis. *Sci Adv* 9. doi: 10.1126/sciadv.ade5079.

Hromada, S., Qian, Y.L., Jacobson, T.B., Clark, R.L., Watson, L., Safdar, N., et al. (2021), Negative interactions determine *Clostridioides difficile* growth in synthetic human gut communities. *Mol. Syst. Biol.* 17. doi: 10.15252/msb.202110355.

Chi, M., Ma, K., Wang, J., Ding, Z., Li, Y., Zhu, S., et al. (2021), The Immunomodulatory Effect of the Gut Microbiota in Kidney Disease. *J Immunol Res* 2021, Review. doi: 10.1155/2021/5516035.

Jia, J., Bao, P., Yu, Q., Li, N., Ren, H., Chen, Q., et al. (2025), *Lactobacillus* Re-Engineers Gut Microbiota to Overcome *E. coli* Colonization Resistance in Mice. *Vet Sci* 12, Article. doi: 10.3390/vetsci12050484.
